# Experimental VLP vaccine displaying a furin antigen elicits production of autoantibodies and is well tolerated in mice†

**DOI:** 10.1039/d4na00483c

**Published:** 2024-10-09

**Authors:** Vili Lampinen, Markus J. T. Ojanen, Fernanda Muñoz Caro, Stina Gröhn, Minna M. Hankaniemi, Marko Pesu, Vesa P. Hytönen

**Affiliations:** a Faculty of Medicine and Health Technology, Tampere University Tampere Finland vesa.hytonen@tuni.fi; b Fimlab Laboratories Ltd FI-33014 Tampere Finland

## Abstract

Proprotein convertase (PCSK) enzymes serve a wide range of regulatory roles in mammals, for example in metabolism and immunity, and altered activity of PCSKs is associated with disorders, such as cardiovascular disease and cancer. Inhibition of PCSK9 activity with therapeutic antibodies or small interfering RNAs is used in the clinic to lower blood cholesterol, and RNA interference -based silencing of *FURIN* (*PCSK3*) is being evaluated in clinical trials as a cancer treatment. Inhibiting these proteins through vaccine-induced autoantibodies could be a patient-friendly way to reduce the frequency of intervention and the overall price of treatment. Here, we show that a self-directed immune response against PCSK9 and furin can be generated in mice by presenting fragments of the proteins on norovirus-like particles (noro-VLPs). We genetically fused three PCSK peptides and the P domain of furin to the SpyCatcher linker protein and covalently conjugated them on noro-VLPs *via* SpyCatcher/SpyTag linkage. Both PCSK9 peptides and the furin P domain generated antigen specific IgGs even without conventional adjuvants. Importantly, vaccinating against furin did not cause adverse events or immune-mediated inflammatory disease. This study adds further support for the feasibility of VLP-based anti-PCSK9 vaccines and shows that the same principles can be applied to make novel vaccine candidates against other endogenous proteins such as furin. We also demonstrate that the noro-VLP can be used as a vaccine platform for presenting self-antigens.

## Abstract 2

A nanoparticle-based vaccine against furin generated autoantibodies in mice.

## Graphical abstract

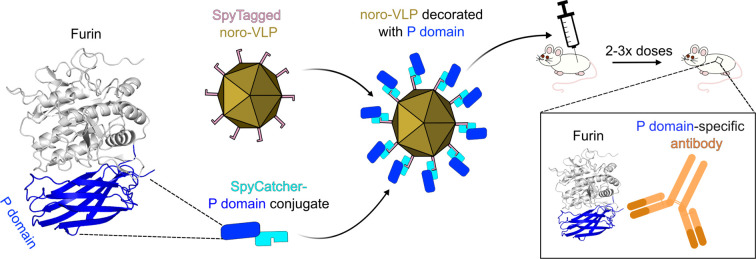

## Introduction

Proprotein convertase subtilisin/kexins (PCSKs) are serine endoproteases that activate, or more rarely, inactivate their substrate proteins *via* post-translational proteolytic cleavage. The conventional PCSKs (PCSK1–7) share a common polybasic cleavage motif ((R/K)–(X)_*n*_–(R/K)↓; X = any amino acid (aa), *n* = 0, 2, 4, or 6 amino acid residues) that enables them to process substrates ranging from secreted hormones to bacterial toxins.^1^ Although the enzymatic functions of conventional PCSKs are partially redundant, their varying expression levels in different tissues and diverse subcellular localizations create major biological differences.^2–4^ For example, *Pcsk2* knock-out (KO) mice are phenotypically normal at birth,^5^ but the germ-line deletion of *Furin*, also known as *Pace* or *Pcsk3*, leads to death at approximately 11 days after fertilization.^6^ In comparison to PCSKs 1–7, PCSK8 (also known as MBTPS1) and PCSK9 have different cleavage site requirements; RX–(L/I/V)–X↓ (X is any aa except Pro, Cys) and (L/I/V)FAQ↓, respectively.^3^ While PCSK8 regulates processes like fatty acid homeostasis by cleaving *e.g.* sterol-regulated element binding proteins (SREBPs), PCSK9 only has autocatalytic protease activity, and its main function is to direct low-density lipoprotein receptor (LDLR) for degradation.

*FURIN* is a well-conserved member of the *PCSK* family that is expressed ubiquitously in all vertebrates.^7^ More than a hundred furin target proteins have been identified in the mammalian proteome,^8^ and many bacterial toxins and viruses such as cholera and diphtheria toxins, influenza and severe acute respiratory syndrome coronavirus 2 (SARS-CoV-2) depend on furin-mediated cleavage for their pathogenicity.^9^ Furin-inhibiting antibodies and small-molecule drugs have been shown to protect against anthrax toxin^10^ and to prevent viral glycoprotein maturation *in vivo*.^11^ In addition, aberrant *FURIN* gene expression or enzyme function is associated with human malignancies, such as lung and skin cancers,^9,12^ and autoimmune diseases, including Sjögren's syndrome and rheumatoid arthritis.^13,14^ In fact, Vigil RNA interference therapy, which inhibits *FURIN* expression using a short hairpin RNA (shRNA) construct, is currently in clinical trials as a regimen for several cancers.^15^ The progression of these cancers is associated with increased concentration of active TGF-β, that is mainly activated by furin-mediated proteolysis. Because of the importance of furin in both transmissible and noncommunicable human diseases, inhibition of furin has significant therapeutic potential.

Vaccines are successfully utilized to fight against infective agents. Recently, experimental vaccines targeting endogenous human proteins have also been proposed to treat *e.g.* cancer and hypertension.^16,17^ While self-directed vaccines have long been unfeasible, conjugating endogenous target protein domains or smaller fragments to an immunogenic carrier, like a virus-like particle (VLP), can be used to overcome peripheral tolerance for the self-antigens.^18,19^ Presentation of an antigen on the surface of a VLP leads to multivalent antigen display, which is needed for T-cell-independent B-cell response against self-antigens through B cell receptor crosslinking.^20^

Our previous studies on norovirus-like particles (noro-VLPs) have revealed that they are particularly robust and easy to modify, produce and store.^21,22^ Noro-VLPs consist of 60, 180 or 240 repeats of the single capsid protein, VP1, depending on the genotype and buffer conditions.^23–26^ VP1 proteins assemble to form the noro-VLP so that the C-terminally fused SpyTag is available for conjugation on the particle surface.^22^ Consequently, we can irreversibly conjugate the SpyTagged noro-VLPs with different kinds of SpyCatcher-fused antigens simply by mixing the components in solution, which makes it straightforward to generate novel experimental vaccines.^27^ As a beneficial “side-product” of the immunization against target antigen, strong immunization against norovirus is generated,^28^ which could be exploited to prevent norovirus infections.

In this study, we evaluated if the noro-VLP platform enables immunization against self-antigens, namely PCSK9 and furin, without severe side effects. For this purpose, we decorated noro-VLPs with two previously published PCSK9-based peptides,^29^ as well as with a furin peptide with pan-PCSK specificity and the P domain of furin. We characterized both B and T cell responses as well as serum cytokines of BALB/c mice immunized with the noro-VLP-protein conjugates. Importantly, immune responses against the endogenous proteins were generated, and the mice showed no signs of immune-mediated inflammatory disease or other adverse effects.

## Materials and methods

### Production of decorated noro-VLP vaccine candidates

SpyTag-noro-VLPs were produced and purified as described previously.^22^ Briefly, baculovirus (*Autographa californica* multiple nuclear polyhedrosis virus) containing the target gene was used to infect Hi5 cells (Thermo Fisher Scientific, USA, #B85502). After 4–6 days of SpyTag-noro-VLP expression, the supernatant was collected and ultracentrifuged using a 30% sucrose cushion. Residual baculovirus was separated from SpyTag-noro-VLP by anion exchange chromatography using pre-packed 5 mL HiTrap Q XL anion exchange column (GE Healthcare, USA, #17-5159-01) at pH 7.0 and the ÄKTA Purifier instrument (Danaher, USA).

SpyCatcher-PCSK expression plasmids were modified from the plasmids described earlier^22^ by replacing the antigen fused to SpyCatcher through restriction-ligation cloning (Genscript, USA). It should be noted that the original version of SpyTag^30^ and the truncated (84 amino acids) version of SpyCatcher^31^ were used in this study (ESI Fig. 1A and B†). While SpyCatcher-fused PCSK-peptides (**PEED**GTRFHRQASK, **DIIG**ASSDCSTC and **SWG**PEDDGKTVDGP) (Addgene IDs 201195, 201196 and 201194, respectively) were produced in *E. coli* (BL21) using a previously established protocol,^22^ SpyCatcher-P domain fusion (Addgene ID 201193) was expressed in *E. coli* C41(DE3) (Lucigen, USA); pre-culture to OD_600nm_ of 0.6–0.8 at +37 °C, gene induction by 1 mM IPTG and overnight culture at +18 °C. The peptides/proteins were purified using HisTag affinity chromatography, dialyzed into PBS buffer and analyzed for purity by SDS-PAGE, followed by concentration determination using the BCA (Thermo Fisher Scientific, USA, #23252) method. The proteins were flash-frozen and stored at −80 °C.

Conjugation of SpyCatcher-antigens on SpyTag-noro-VLP was done by mixing them together in a 2 : 1 (P domain) or 4 : 1 (peptides) molar ratio, allowing ∼40 hours of reaction time at +4 °C and then removing unreacted SpyCatcher-antigen by dialysis through a 1000 kDa cut-off membrane. For the first immunization experiment, the vaccine candidates were conjugated and dialyzed immediately before the first immunization and stored at +4 °C for the three weeks between the immunizations. For the second immunization experiment, we conjugated the SpyCatcher-P domain on SpyTag-noro-VLP as described above but used size-exclusion chromatography with the ÄKTA Purifier instrument and HiPrep 16/60 Sephacryl S-500 HR column (Cytiva, USA, #28935606) to remove nonconjugated antigen. The vaccine candidates were aliquoted and frozen. On the day of immunization, an aliquot of each vaccine candidate was thawed. The integrity of the antigen was verified by dynamic light scattering (DLS) and SDS-PAGE from a test aliquot after a freeze-thaw round.

### Characterization of purified proteins and particles

Protein purity and conjugation efficiency were estimated by densitometry analysis of Stain-free (Bio-Rad, USA) or silver stained SDS-PAGE gels with the Image Lab software (Bio-Rad). For Western blotting, we transferred the proteins onto nitrocellulose membrane using Trans-blot Turbo (Bio-Rad). The SpyCatcher-PCSK fusion proteins were identified based on the binding of an HisTag antibody (1 : 10 000, Thermo Fisher Scientific, #ma1-21315). A mouse monoclonal gp64 antibody (1 : 2000, Santa Cruz Biotechnology, USA, #sc-65499) was used to confirm the absence of baculovirus in the purified VLPs, using a high-titer baculovirus stock solution as a positive control. The bound primary antibodies were visualized by IRDye 800CW goat anti-mouse IgG secondary antibody (1 : 20 000, LI-COR Biosciences, USA, #926-32210) and the Odyssey CLx instrument (LI-COR Biosciences, USA). Endotoxin concentrations were determined with Pierce LAL Chromogenic Endotoxin Quantitation Kit (Thermo Fisher Scientific, #88282) and the amount of residual DNA was measured with the Quant-iT dsDNA high sensitivity kit (Thermo Fisher Scientific, #Q33120). We used dynamic light scattering (DLS) analysis with the Zetasizer Nano ZS (Malvern Instruments, UK) to measure the size and polydispersity of produced nanoparticles and proteins. F200 S/TEM (Jeol, Japan) transmission electron microscope (TEM) was used to examine the morphology of conjugated noro-VLPs after negative staining with 1% uranyl acetate.

### Immunizations

In the first immunization experiment, female BALB/c OlaHsd mice (Envigo, the Netherlands), were randomly divided into 6 groups (Table 1, 7 mice per group) and acclimatized for a week before the experiment. At the time of the first immunization (day 0), the mice were 8 weeks old. The animals were immunized intramuscularly (i.m.) at the caudal thigh muscle under inhalation anesthesia by isoflurane (Attane vet, Vet Medic Animal Health, Finland, #AP/DRUGS/220/96) at days 0 and 21. The antigen doses (Table 1) were calculated by multiplying total protein dose with the conjugation efficiency and considering the size of the antigen compared to fused SpyCatcher and noro-VLP used in antigen conjugation. External adjuvants were not included in the vaccine formulations. Whole blood was collected at the time of sacrifice (day 35) using cardiac puncture, and the serum was separated with blood collection tubes (Thermo Fisher Scientific, #365968) following manufacturer's instructions and frozen until analysis. The spleens were collected after sacrifice for splenocyte extraction.

**Table 1:** Dosage of the vaccine components in the immunization experiments. The table uses the following abbreviations: SpyCatcher (SC), SpyTag-noro-VLP (NV), and furin P domain (Pdom)

Vaccine group	Total protein/dose (μg)	Conjugation efficiency (%)	Total antigen/dose (μg)	Conjugated NV/dose (μg)
**1st immunization experiment**				
NV-PEED	19	50	0.2	9.3
NV-DIIG	19	50	0.1	9.3
NV-SWG	19	50	0.2	9.3
NV-P domain	46	20	3.1	9.2
NV-SpyTag	19	n/a	0	0
PBS	0	n/a	0	0

**2nd immunization experiment**				
NV-P domain + AlOH	20	5	3.7	1.0
NV-P domain/SC-P domain + AlOH^a^	20	5	3.7	1.0
SC-P domain + AlOH	6.7	n/a	3.7	0
NV-SpyTag + AlOH	20	n/a	0	0
TBS + AlOH	0	n/a	0	0

a This group received P domain conjugated on noro-VLP on the first two immunizations and nonconjugated SpyCatcher-P domain on the last two immunizations.

In the second immunization experiment, female BALB/cJRj mice (Janvier Labs, France) were divided into 5 groups (Table 1, 5 mice per group) and acclimatized for a week. The mice were 6 weeks old at the first immunization. The animals were immunized subcutaneously (s.c.) over the shoulders at days 0, 21, 42 and 63. The antigen doses were calculated as earlier, but now we added 100 μg “Alhydrogel adjuvant 2%” (Invivogen, USA, #vac-alu-250) Al(OH)_3_ per dose. The mice were sacrificed for the isolation of blood and spleen on day 76, as described previously.

The pre-clinical experiments were approved by the Finnish National Experiment Board (Permission numbers ESAVI/6781/2018 and ESAVI/16254/2019). All efforts were made to minimize animal suffering and to reduce the number of animals used. The welfare of the animals was monitored throughout the first immunization experiment through weekly weighing and at procedures in the second experiment. Additionally, Animal Research: Reporting of *In Vivo* Experiments (ARRIVE) guidelines were followed.

### Antibody titration

The amounts of total IgG antibodies against SpyTag-noro-VLP, recombinant human furin (Peprotech, UK, #450-47) and recombinant mouse PCSK9 (Abcam, UK, #ab167759) were measured from mouse serum samples, as described earlier.^32^ Briefly, Maxisorp 96-well-plates (Thermo Fisher Scientific, USA, #439454) were coated with 50 ng of SpyTag-noro-VLP, furin or PCSK9. After blocking and IgG binding steps, antigen-bound IgGs were detected with horseradish peroxidase conjugated horse anti-mouse antibody (1 : 4000, Vector, USA, #PI-2000) and OPD substrate (Merck, USA, #P8412). Optical densities at 490 nm (OD_490_) were measured with a microplate reader (Victor^2^, PerkinElmer, USA). Endpoint titers were defined as the reciprocal of the highest serum dilution with an OD_490_ above the positivity cut-off value. The positive cut-off value was defined as the (mean absorbance) + *X* × (standard deviation) of negative control group sera at the lowest dilution measured, or at least 0.07, which is the maximum background absorbance in buffer sample. *X* is a factor that depends on the desired confidence level and the number of mice in the control group. We used factors of 2.077 or 2.335 to reach confidence levels of 95% with 7 or 5 mice in the negative control groups in the experiments.^33^

### Splenocyte activation

The spleens of mice were collected and disrupted by crushing, and the splenocytes were strained through 70 μm Corning strainers (Sigma-Aldrich, USA) to obtain single cell suspensions. Erythrocytes were lysed using 2 mL of ACK lysis buffer (Thermo Fisher Scientific) and the cells counted manually using Bürker chambers or with LUNA-FL cell counter (Logos Biosystems, South Korea). Splenocytes were on ice or at +4 °C during the isolation procedure. For activation, a maximum of 2.5 × 10^6^ cells were seeded onto 24-well culture plates with 1 mL of RPMI-1640 media (Lonza, Switzerland), supplemented with 10% FBS (Thermo Fisher Scientific), 1% penicillin-streptomycin (Lonza), 1% l-glutamine (Lonza) and 2-mercaptoethanol (50 μM), and stimulated for 3 days at 37 °C using 5 μg of soluble anti-CD3 monoclonal antibody (17A2; positive control) (Thermo Fisher Scientific), recombinant mouse PCSK9 (Abcam, UK, #ab167759), SpyTag-noro-VLP, SpyCatcher-PEED, SpyCatcher-DIIG, SpyCatcher-SWG, or SpyCatcher-P domain. Following stimulation, the culture supernatants were collected and frozen at −20 °C or −80 °C.

### Flow cytometry

The splenocytes were extracted as described above and 3.0 × 10^6^ of cells were transferred onto 24-well plates in 1 mL of RPMI-1640 media (supplemented as described above), and treated with phorbol 12-myristate 13-acetate (PMA, 50 ng mL^−1^) (Sigma-Aldrich), calcium ionophore (1 μg mL^−1^) (Sigma-Aldrich), GolgiPlug (1.0 μL mL^−1^) and GolgiStop (0.7 μL mL^−1^) (Becton, Dickinson and Company) for 4 hours at +37 °C. Subsequently, cells were washed with media and stored at +4 °C for 18 hours. Before antibody staining, the splenocytes were incubated with the FVS510 viability stain (Becton, Dickinson and Company) and treated with a CD16/CD32 monoclonal antibody solution (Thermo Fisher Scientific or Becton, Dickinson and Company) for Fc-receptor blockade. Cell surface staining was performed using anti-CD3-FITC, anti-CD8a-PerCP-Cy5.5, anti-CD4-APC-Cy7 and anti-CD19-BV786 antibodies (Thermo Fisher Scientific), whereas Cytofix/Cytoperm kit (Becton, Dickinson and Company) and anti-IFN-γ-PE-Cy7 and anti-TNF-PE-Cy7 antibodies (Thermo Fisher Scientific) were used for intracellular staining. Of note, since both anti-IFN-γ and anti-TNF antibodies were conjugated to PE-Cy7, samples were divided into two tubes before their intracellular staining. Individually stained primary cells and an anti-rat and anti-hamster Igκ/negative control compensation particles set (Becton, Dickinson and Company) were utilized for compensation. CytoFLEX (Beckman Coulter Life Sciences, USA) was used for flow cytometry.

### Serum protein concentrations

The furin concentrations from mouse sera were measured with furin ELISA kit (#E9700m, EIAab, Wuhan, China), according to the manufacturer's instructions. We used the V-PLEX Plus Proinflammatory Panel 1 Mouse Kit (#K15048G, Meso Scale Discovery, USA) to measure cytokine concentrations in the freeze-stored serum samples of immunized and control mice from both immunization experiments. The measurement was executed according to kit instructions, using serum samples diluted 1 : 2 in Diluent 41 from the kit. A single mouse serum sample from the PBS group was diluted 1 : 4.2 due to low sample availability. While 16% (10/64) of the serum samples were measured as technical duplicates, the rest of the samples were quantified only once. The standard deviation of the assay was determined as 5.8 ± 9.6% of the mean concentration.

### Statistical analyses

For statistical analyses, we used GraphPad Prism version 8.3.0 and defined that *p* < 0.05 indicates statistically significant difference. To estimate differences in end-point titers, cytokine quantities, and cell frequencies between vaccine and control groups, we used Kruskal–Wallis followed by Dunnet/Dunn's test. To compare the mean cytokine concentrations in mouse blood samples, we used ordinary one-way ANOVA followed by Tukey's test. The group sizes for immunizations were chosen based on previous experimental observations.^22,28^

## Results

### Production of SpyCatcher-PCSK fusion proteins and their conjugation on the noro-VLP

**Fig. 1:**
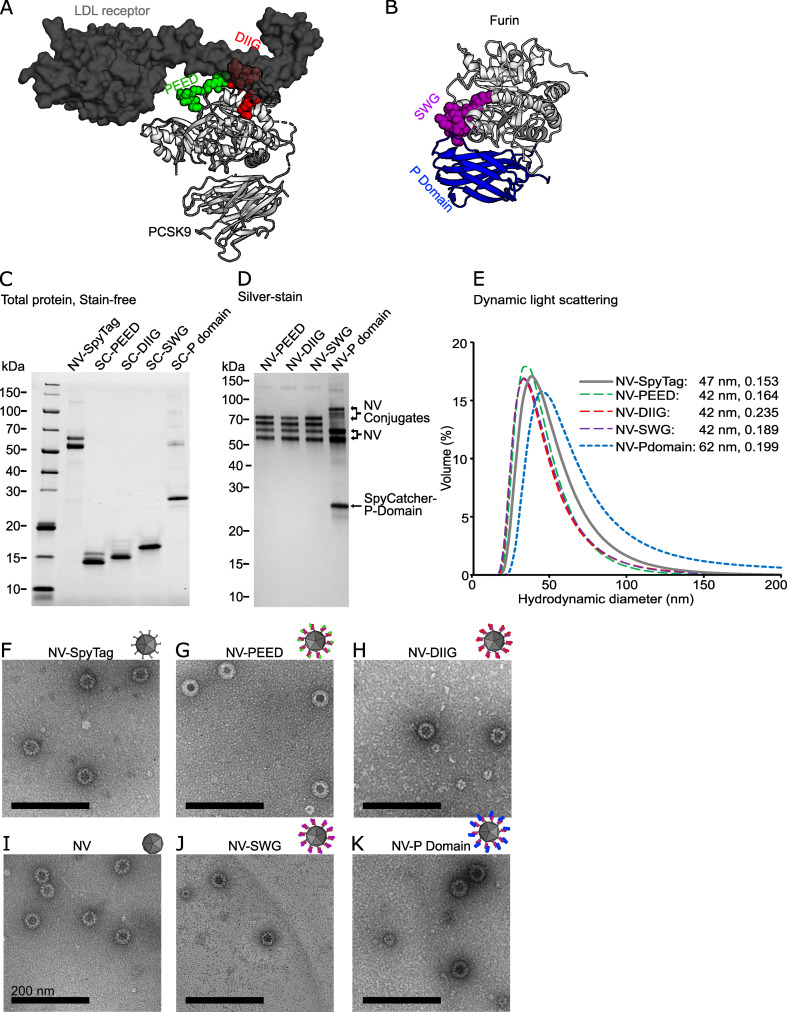
Design and production of the noro-VLP (NV) -based vaccine candidates against PCSK9 and furin. (A) A visualization of a co-crystal structure of PCSK9 bound to a fragment of the LDL receptor. The targeted regions of PCSK9 are located at the receptor binding site and are highlighted with green (PEED) and red (DIIG). (B) A visualization of a crystal structure of furin. The SWG peptide is located at the active site and is highlighted with purple. Furin P domain is shown in blue. (C) Stain-free SDS-PAGE gel of representative vaccine components before conjugation. NV is visible as a double band due to a partial N-terminal cleavage described earlier.^34^ Lanes contain ∼1–2.5 μg of protein. (D) Silver-stained SDS-PAGE gel of the SpyTag/SpyCatcher conjugated vaccine candidates after removal of nonconjugated recombinant proteins by dialysis. All lanes contain ∼500 ng of protein. (E) Dynamic light scattering volume distributions of the vaccine candidates. The hydrodynamic diameter peaks (nm) and polydispersity indexes are also listed in the figure. (F–K) Transmission electron micrographs of NV, NV-SpyTag and conjugated NV-PCSK9 (G and H) and NV-furin (J and K) constructs at a 600 00× magnification. The black scale bars are 200 nm. The following Protein Data Bank structures were used in visualizing the protein structures: LDL receptor bound PCSK9: 3M0C, Furin: 5JMO. The uncropped gels are shown in ESI Fig. 3.†

We have previously established a modular noro-VLP vaccination platform using the SpyCatcher/SpyTag-conjugation system.^22^ To produce PCSK-targeting vaccine candidates, we chose two regions of PCSK9 (abbreviated PEED and DIIG) from the LDL receptor binding site (Fig. 1A),^29^ a region near the PCSK active site (SWG) that is more than 85% conserved between the conventional PCSKs 1–7 (Fig. 1B and ESI Fig. 2A†) as well as the furin-specific P domain of furin (residues 444–576) (Fig. 1B). Fully conserved sequences between the corresponding mouse and human *PCSK* genes were chosen for the peptide antigen production. Additionally, only 5% (7/133) of the amino acids in the furin P domain construct differ between the mouse and human protein (ESI Fig. 2B†), indicating strong conservation.

The peptides (PEED, DIIG, SWG) were expressed to a high yield in *E. coli* as SpyCatcher fusions, and more than 50 mg of >90% pure protein could be obtained per one liter of culture as assessed by BCA and densitometry analyses (Fig. 1C and ESI Fig. 3†). Matrix-assisted laser desorption/ionization mass spectrometry (MALDI-MS) further confirmed that the SpyCatcher-peptides were expressed in full (ESI Fig. 4†). Furin P domain proved to be a more challenging protein for the *E. coli* expression system. In fact, when expressed alone, P domain was insoluble (data not shown). However, adding the SpyCatcher moiety to the N-terminus of the domain improved the solubility and reasonable yields were obtained (∼1 mg of >90% pure protein per liter of culture). For SpyCatcher-P domain, SDS-PAGE and western blotting were used to ensure the molecular weight and the integrity of the antigen (Fig. 1C and ESI Fig. 3†).

To produce the final vaccine candidates, we decorated the insect cell produced SpyTag-noro-VLPs with SpyCatcher-PCSK fusion proteins as described previously.^22^ Based on SDS-PAGE densitometry, the conjugation efficiencies were approximately 50% with PEED, DIIG and SWG peptide vaccines, and 20% with the P domain vaccine (Fig. 1D, Table 1 and ESI Fig. 3†). After conjugation, nonconjugated SpyCatcher-antigen was removed by dialysis with a 1000 kDa cutoff membrane. While high homogeneity (>99%) was achieved for the noro-VLP-PEED, noro-VLP-DIIG and noro-VLP-SWG vaccines, the noro-VLP-P domain sample contained an approximately equal amount of covalently and non-covalently bound SpyCatcher-P domain after dialysis or SEC (Fig. 1D and ESI Fig. 3†), suggesting dimerization of P domain and attachment on VLP *via* a single subunit of the dimer. All vaccine candidates contained <0.6 EU μg^−1^ of endotoxins and <11 ng of DNA per vaccine dose.

According to DLS analysis, the mean hydrodynamic diameters of noro-VLP-PEED, noro-VLP-DIIG and noro-VLP-SWG were smaller than that of SpyTag-noro-VLP, whereas the largest diameter was seen with the noro-VLP-P domain (Fig. 1E). Correspondingly, while an expected icosahedral morphology typical to noro-VLPs were observed with all the vaccine candidates using transmission electron microscope (TEM), an increased frequency of the 60-meric noro-VLP form was seen in the noro-VLP-PEED, noro-VLP-DIIG and noro-VLP-SWG batches (Fig. 1G, H, and J). In noro-VLP-P domain and SpyTag-noro-VLP, the 60-meric form is in minority (<10%), but in the peptide-SpyCatcher-conjugated vaccine batches, the 60-meric form is more common than the bigger particle species. More thorough structural studies are required to conclude if this difference is due to differences in the conjugation partner, or *e.g.* in pH differences in purification that would cause dynamic changes in stoichiometry.^24^

### SpyCatcher-PCSK fusion proteins presented on norovirus-like particles induce autoantibodies in mice

**Fig. 2:**
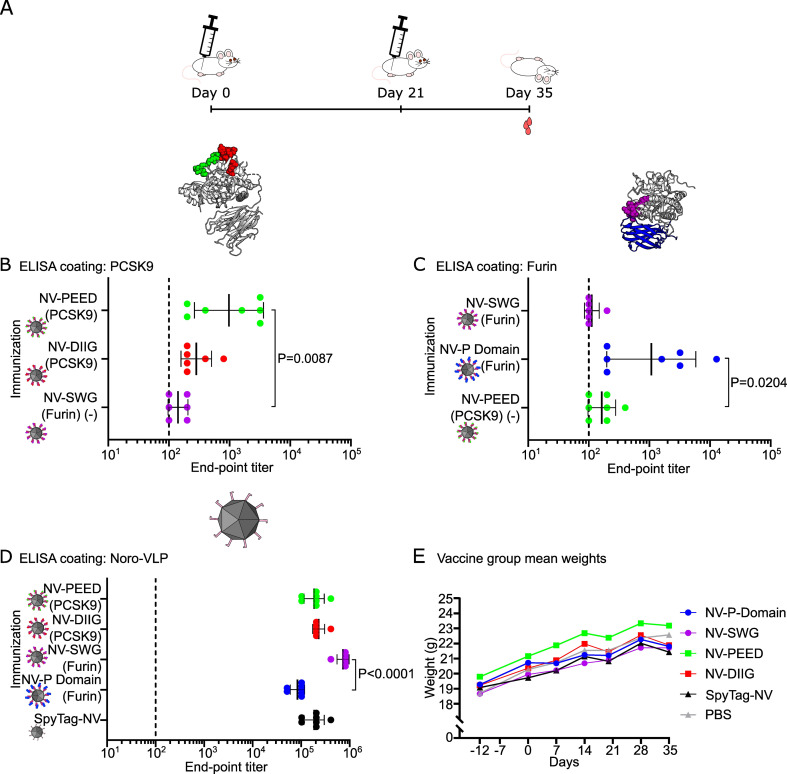
Unadjuvanted PCSK-coated noro-VLPs (NV) induce the production of PCSK9- and furin-specific IgGs. (A) A schematic overview of the immunization experiment. 8 week-old BALB/c mice (*n* = 6, all females) were injected twice i.m. with PCSK-decorated NVs, SpyTag-NV or PBS at days 0 and 21. On day 35 the animals were euthanized, and blood and spleen collected for analysis. (B–D) End-point antibody titers were measured using ELISA. Plates were coated with the corresponding protein and the IgG antibody titers against recombinant PCSK9 (B), recombinant furin (C), and SpyTag-NV (D) are shown. The end-point titer is the reciprocal of the lowest serum dilution that gives a significantly higher absorbance as compared to 1 : 200 diluted negative control mouse group serum (SpyTag-NV in panels A and C or PBS in panel D). Mean titers ± standard deviation are depicted. Differences in means were tested with Kruskal–Wallis test followed by Dunnet's test and the significant adjusted *P* values are shown. As an additional negative control (−), we used serum from a group that is naive to the tested protein in panels (B) and (C). Undetectable antibody levels were denoted with the reciprocal titer 100 (vertical dashed line). (E) The arithmetic mean weight of mice in different vaccination groups during the experiment is represented as a function of time. Each dot represents a single mouse in panels (B)–(D).

Previous studies have shown that experimental vaccines displaying PCSK9 self-antigen peptides on bacteriophage VLPs can elicit high-titer IgG responses against PCSK9 in mice and macaques.^29,34^ Here, we prepared two previously tested peptide antigens from PCSK9 (PEED and DIIG), an analogous peptide from the active site of furin (SWG) and the whole furin P domain for display on the noro-VLP *via* SpyCatcher/SpyTag linkage. To study the immunogenicity of the PCSK-decorated noro-VLPs, we immunized BALB/c mice twice i.m. with unadjuvanted antigen-conjugated noro-VLP (19–46 μg), SpyTag-noro-VLP (19 μg) or PBS with a three-week interval between the immunizations and collected their blood and spleens 35 days after the start of the experiment (Table 1 and Fig. 2A).

Both PCSK9 peptide vaccines produced detectable amounts of anti-PCSK9 IgG antibodies with geometric mean titers (GMTs) of 280 in noro-VLP-DIIG vaccinated mice and 980 in the noro-VLP-PEED group (Fig. 2B). Moreover, the anti-PCSK9 IgG titers in the noro-VLP-PEED vaccinated mice were significantly higher than in the negative control group of noro-VLP-SWG (*P* = 0.0087). The conserved SWG peptide, located at the active site of conventional PCSKs (*e.g.* that of furin), generated no detectable furin-specific antibodies, whereas significantly higher anti-furin IgG titers were detected in the noro-VLP-P domain vaccinated group (GMT = 1100) in comparison to the corresponding control group of noro-VLP-PEED (GMT = 160, *P* = 0.0204) (Fig. 2C). The response in the furin P domain group was heterogeneous; 3 mice out of 7 responded poorly (GMT < 300), whereas one immunized mouse reached a titer of 1.3 × 10^4^ furin-specific IgG.

All vaccinated mice produced high noro-VLP-specific IgG titers, with the highest titers measured in the animals vaccinated with noro-VLP-SWG (GMT = 7.3 × 10^5^), which was significantly more than in P domain-noro-VLP group (GMT = 8.4 × 10^4^, *P* < 0.0001) (Fig. 2D). While the weight of all the mice increased steadily during the experiment (Fig. 2E), one mouse of the PCSK9-DIIG group died a few days before the predetermined termination date. Prior to its death, the mouse did not show any health issues or visible differences in appearance in comparison to the control group animals or its cage mates.

As described earlier, furin-specific antibodies have been demonstrated to inhibit furin-mediated activation of diphtheria toxin.^35^ To this end, we used serum from the SpyCatcher-P domain injected mice that had the highest furin-specific IgG titers and incubated it with diphtheria toxin and recombinant furin. However, we did not see measurable difference in furin inhibition activity between treatment and control groups (data not shown).

### Splenocytes extracted from immunized mice elicit an IL-2 and IFN-γ recall response against noro-VLP and SpyCatcher-P domain

**Fig. 3:**
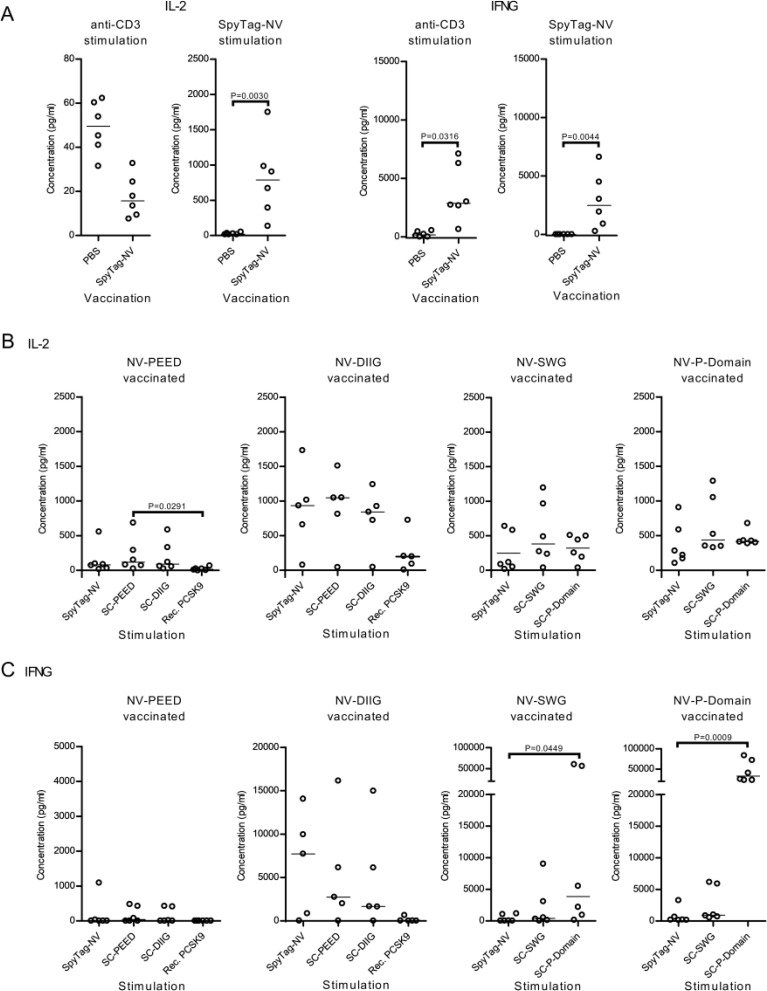
Antigen-specific SpyTag-noro-VLP (NV) and furin P domain IFN-γ responses are elicited upon re-stimulation with the cognate proteins. Splenocytes were isolated from BALB/c mice (*n* = 5–6, all females) vaccinated with PBS, SpyTag-NV or PCSK-conjugated NV, and the cells were stimulated *in vitro* for 3 days at 37 °C. The culture media was collected at 3 dps and the IL-2 and IFN-γ (IFNG) concentrations quantified using ELISA. (A) Splenocytes from SpyTag-NV and PBS injected mice were stimulated using anti-CD3 antibody or SpyTag-NV. In (B) and (C) splenocytes from mice vaccinated with NV-PEED and NV-DIIG from PCSK9 and NV-SWG and NV-P domain from furin were stimulated with the described antigens. Scatter dot plots with median are shown for each group. Nonparametric Kruskal–Wallis one way ANOVA followed by Dunnet/Dunn's test was used for the statistical evaluation of differences. SpyCatcher is abbreviated as SC in the figure.

To measure the vaccination-induced memory T cell responses, we used splenocytes from the immunized mice, re-stimulated them with vaccine components *in vitro* and measured IFN-γ and IL-2 levels from the culture supernatant 3 days post stimulation (dps) using ELISA. We also used anti-CD3 antibodies as an unspecific T-cell receptor mediated control stimulation. Here, SpyTag-noro-VLP stimulation caused higher IL-2 (*P* = 0.0030) and IFN-γ (*P* = 0.0044) levels in the noro-VLP-vaccination group in comparison to splenocytes obtained from the PBS vaccinated animals (Fig. 3A, ESI Fig. 5A and B†), suggesting a strong recall T cell response against noro-VLP in the immunized mice. In line with this, restimulation of the splenocytes from the noro-VLP-vaccinated mice using anti-CD3 antibodies caused increased IFN-γ production compared to PBS control cells (*P* = 0.0316).

To specifically determine the extent of the antigen-specific T cell responses, we next compared the IL-2 and IFN-γ concentrations from samples within each vaccination group (Fig. 3B, C and ESI Fig. 5C†). Here, stimulating furin P domain immunized cells with recombinant SpyCatcher-P domain caused significantly higher IFN-γ production in comparison to SpyTag-noro-VLP stimulated cells (33 000 *vs.* 910 pg mL^−1^, *P* = 0.0009) (Fig. 3C), whereas antigen-specific IL-2 or IFN-γ responses were not observed in other vaccination groups. Suggesting that also the SpyCatcher linker is immunogenic, stimulating splenocytes with SpyCatcher-conjugated antigens caused IL-2 and IFN-γ levels that were comparable with SpyTag-noro-VLP stimulation (Fig. 3B and C). Furthermore, while recombinant PCSK9 caused low or non-existing cytokine response in the splenocytes of the noro-VLP-PEED and noro-VLP-DIIG vaccination groups (Fig. 3B and C), unspecific IFN-γ production was observed in furin-P domain stimulated cells of the SpyTag-noro-VLP and noro-VLP-SWG vaccination groups (Fig. 3C and ESI Fig. 5C†).

All in all, while our results suggest antigen-specific recall responses against SpyTag-noro-VLP, SpyCatcher and SpyCatcher-P domain, targeted T cell responses against PCSK9 could not be detected. Moreover, the P domain of furin seem to cause non-specific immunostimulation in splenocyte cultures.

### Adjuvanted immunization with furin P domain elicits anti-furin antibody production, but does not induce immune-mediated inflammatory disease

**Fig. 4:**
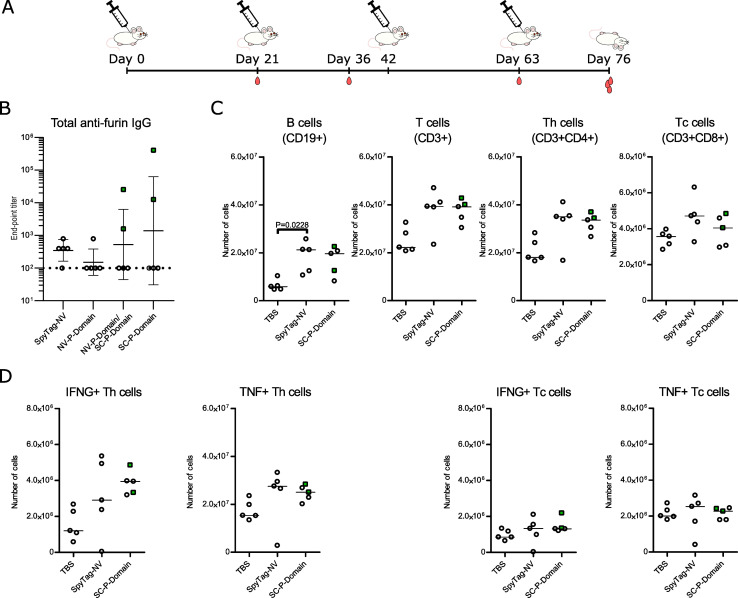
An adjuvanted immunization with furin P domain yields high anti-furin titers in individual mice but causes no differences in lymphocyte populations or aberrant T cell activation. (A) A schematic overview of the immunization experiment. BALB/c mice (*n* = 5, all females) were injected four times s.c. with alum adjuvanted P domain vaccine candidates, SpyTag-noro-VLP (NV), or TBS at days 0, 21, 42, and 63. Tail vein blood samples were collected at days 21, 36, 63, and on day 76 the animals were euthanized, and blood and spleen collected for analysis. (B) Antibody titers at day 76 were measured using ELISA. Plates were coated with recombinant furin and the furin-specific IgG antibody titers are shown. The end-point titer is the reciprocal of the lowest serum dilution that gives a significantly higher absorbance as compared to negative control (TBS) mouse group serum diluted 1 : 400. Mean titers ± standard deviation are depicted. Each dot represents a single mouse. Undetectable antibody levels were denoted with the reciprocal titer 100 (vertical dashed line). (C–D) Flow cytometric evaluation of lymphocyte populations (C) and the IFN-γ- (IFNG-) and TNF-producing T helper (Th) and T cytotoxic (Tc) cells from the spleen (D). The numbers of cells are based on the frequency within the live gate and the total splenocyte count. Target populations were identified based on their expression of CD19, CD3, CD4, CD8, IFN-γ and TNF. The gating strategies in flow cytometry are shown in ESI Fig. 7.† Nonparametric Kruskal–Wallis one way ANOVA followed by Dunnet/Dunn's test was used for the statistical evaluation of differences. Throughout the figure, mice showing >10^3^ anti-furin titers were indicated with green color and rectangular symbols.

To obtain stronger antibody responses against furin and to further investigate the safety of furin-targeted immunization, we conducted another immunization experiment with the furin P domain using an adjusted protocol. More specifically, we used unconjugated and noro-VLP-conjugated P domain (Table 1), included an adjuvant to all groups and performed four immunizations within the 76 day experiment (Fig. 4A). Surprisingly, the noro-VLP conjugated furin P domain now failed to produce detectable anti-furin antibodies by day 36 (ESI Fig. 6A†), and only one mouse developed anti-furin antibodies by day 76 (Fig. 4B). Conversely, noro-VLP specific IgGs were detected in both noro-VLP and noro-VLP-P domain vaccinated mice already after first immunization at 21 days (ESI Fig. 6B†). By day 76, the group that received noro-VLP-conjugated SpyCatcher-P domain on the first two immunizations and unconjugated SpyCatcher-P domain on the last two had an anti-furin GMT of 530, whereas unconjugated SpyCatcher-P domain generated most anti-furin antibodies with a GMT = 1400 (Fig. 4B). It should be noted that measured conjugation efficiency was lower in the adjuvanted noro-P domain groups than in the experiment without added adjuvants (Table 1).

Even though maximum titers of 2.6 × 10^4^ and 4.1 × 10^5^ were observed in the serum samples of the NV-P domain/SC-P domain group and in the unconjugated SpyCatcher-P domain group, respectively, the IgG titers displayed high variability: the anti-furin response was detected in only one or two mice per group (Fig. 4B). The antibody measurements from different time points additionally show that the anti-noro response decreased from day 63 to day 76, whereas the anti-SpyCatcher response stayed relatively stable, postulating that only the anti-furin response benefitted from the fourth immunization (ESI Fig. 6†).

Vaccinating against an important regulator of cellular functions raises questions about safety. We have previously demonstrated that furin-deficient T cells have an altered activation status and that the KO of furin in myeloid cells alters the cytokine profile in the serum.^36–38^ Consequently, we next studied whether vaccinating against furin could impact splenic lymphocyte frequencies or T cell mediated cytokine production. We determined the frequencies and absolute numbers of CD19^+^ B cells, CD4^+^ and CD8^+^ T cells and IFN-γ and TNF producing T cells as well as the mean fluorescence intensities (MFI) of the IFN-γ and TNF positive populations from the spleens of TBS, SpyTag-noro-VLP and furin-P domain vaccinated mice using flow cytometry (Fig. 4C, D and ESI Fig. 7†). Our analysis indicated significantly increased numbers of B cells in the SpyTag-noro-VLP vaccinated mice in comparison to the TBS controls (*P* = 0.0228) but other statistically significant differences in B cell populations were not observed (Fig. 4C and ESI Fig. 8†). Furthermore, no significant differences were detected in group-wise comparisons of the CD3^+^ T cells or their sub-populations; CD4^+^, CD8^+^, CD4^+^IFNG^+^, CD4^+^TNF^+^, CD8^+^IFNG^+^ and CD8^+^TNF^+^, suggesting that furin-targeting vaccination does not alter lymphocyte proliferation or T cell mediated production of IFN-γ or TNF in comparison to controls.

We also measured IFN-γ, IL-10, IL-12p70, IL-1β, IL-2, IL-4, IL-5, IL-6, KC/gro and TNF cytokine levels from the serum samples of mice from both immunization experiments with a Meso Scale Discovery multiplex assay kit. No significant differences in blood cytokine concentrations were detected between vaccination groups within the same immunization experiment (Additional file 2). However, IL-1β concentrations were systematically higher in all mouse groups of the second immunization experiment, including buffer control (ESI Fig. 9†), which suggests a substantial inflammatory response caused by the Al(OH)_3_ adjuvant. Importantly, we conclude that vaccinating against furin did not cause immune-mediated inflammatory disease in mice.

## Discussion

In the clinic, anti-PCSK9 antibodies (*e.g.* evolocumab, Amgen, USA) and RNA interference therapy (Inclisiran, Alnylam Pharmaceuticals, USA) are used in conjunction with statins in difficult-to-treat cases of high cholesterol, whereas small molecule furin inhibitors have demonstrated efficacy against bacterial toxins and viral infection in preclinical studies.^10,11^ Antibody, RNA interference or small molecule inhibitor therapies may require a lifelong regimen and frequent drug administration by a health care professional, whereas vaccinating against endogenous proteins could theoretically activate the immune system to continuously produce antibodies for up to years after immunization and thereby potentiate, supplement or even replace the drugs administered regularly. In the current study, we immunized mice against endogenous PCSK9 and furin, and demonstrate that a noro-VLP vaccination platform can be used to induce an IgG response against self-peptides and protein domains. We did not detect any adverse effects, such as weight-loss or immune-mediated inflammatory disease, when we targeted the active site of conventional PCSKs (furin-SWG), or furin's P domain by vaccination.

Based on the solved crystal structures of furin^39^ and PCSK9 ^40^ and AlphaFold predictions of PCSKs 1, 2, 4, 5, 6, 7, and 8 (Uniprot IDs P28840, P16519, Q6UW60, Q92824, P29122, Q16549, Q14703),^41,42^ all PCSK enzymes share similar overall fold with a catalytic domain and a recognizable β-barrel P domain. While the catalytic domain of PCSK9 binds the LDL receptor, in conventional PCSKs this domain is expectedly required for enzymatic activity. In turn, the P domain is essential for the proper folding of PCSKs and it has been shown to regulate the activity of the catalytic domain through ionic and hydrophobic interactions.^43,44^ Despite our failed attempts to produce the P domain of furin without a fusion partner (likely caused by the hydrophobic patches on P domain's surface), to our knowledge, the recombinant SpyCatcher-fused furin P domain is the first successful attempt in producing the P domain from any PCSK without the catalytic domain.

It has been previously demonstrated that PCSK9-targeting peptides genetically fused to bacteriophage VLP or keyhole limpet hemocyanin can elicit PCSK9-specific antibody production in mice, rats and in non-human primates.^29,45–47^ Recently, PCSK9 peptides and full-length PCSK9 were conjugated on AP205 bacteriophage VLP *via* SpyCatcher/SpyTag and both generated PCSK9 antibodies in mice.^34^ In line with this, DIIG- and PEED-peptides conjugated to noro-VLP produced detectable IgG titers against PCSK9 (geometric means of 280 and 980, respectively), albeit at lower levels than the corresponding antigens called “368–381” and “207–223” by Crossey *et al.*, with titers higher than 1 × 10^4^. Interestingly however, while the PCSK9-specific titers in the current study were higher than those against influenza M2e peptide achieved with a comparable noro-VLP-based vaccination strategy,^28,48^ no detectable IgGs were present in the serum of the mice vaccinated against the SWG peptide. Further studies are clearly required to demonstrate whether the differences in the peptide-specific IgG titers are due to intrinsic properties of the peptides, the diverse immunization strategies, such as adjuvant usage and numbers of injections, or reflect the properties of the VLP vaccination platform used here.

In our first immunization experiment, the highest antigen-specific IgG titers were achieved in mice vaccinated with furin P domain decorated noro-VLPs. This demonstrates that endogenous furin can be targeted *via* experimental vaccination. While purified anti-furin nanobodies have been reported to inhibit furin-mediated processing of large substrates, like that of diphtheria toxin,^35^ the anti-furin sera was not able to inhibit furin-mediated activation of diphtheria toxin *in vitro*. We cannot, however, rule out diphtheria processing by other serum PCSKs in our serum-based assay. Additionally, since the active site of furin is not localized on the P domain, it is also possible that the P domain-specific antibodies do not directly inhibit the function of the enzyme. Previously, peptide-based vaccines and monoclonal antibodies against PCSK9 have been demonstrated to cause increased PCSK9 serum concentrations,^29,46,47,49^ whereas vaccinations with full-length PCSK9 was reported to lower PCSK9 concentration in the serum.^34^ The earlier was explained by formation of circulating immune complexes and the latter by effective opsonization and system clearance. In both cases, the vaccines and antibodies inhibited PCSK9 function as demonstrated by lowered blood cholesterol levels. To determine whether furin-targeting vaccinations could alter circulating furin levels, we aimed to quantify furin from the serum of immunized mice using ELISA. In our measurements, we however detected correlation between measured furin concentration and the used serum dilution, suggesting that the assay is inhibited by serum components. Consequently, accurate conclusions cannot be drawn from this data.

While cross-reactive and vaccination-induced T cell responses are important in immunity against pathogens including SARS-CoV-2,^50^ T cells are also intricately linked to the activation of other immune cells such as macrophages and B cells.^51^ In contrast, T cells are known to facilitate several autoimmune diseases such as multiple sclerosis, type 1 diabetes and rheumatoid arthritis,^51^ which has raised major safety concerns in relation to self-directed vaccination.^52^ To evaluate the T cell mediated immunity in PCSK immunized mice and the controls, we measured the recall response of antigen stimulated splenocytes by evaluating IFN-γ and IL-2 levels from the culture media. The high cytokine level medians in noro-VLP immunized cells stimulated with SpyTag-noro-VLP argue for a memory T cell specific immune response against noro-VLP. Similarly, splenocyte activation with recombinant SpyCatcher-P domain induced significantly higher IFN-γ production in the noro-VLP-P domain vaccinated mice in comparison to SpyTag-noro-VLP controls, which suggests specificity against the P domain. However, this conclusion should be treated with caution, because of the high IFN-γ production observed also in splenocytes from SpyTag-noro-VLP and noro-VLP-furin-SWG vaccinated groups that were stimulated with the P domain.

In order to boost the antibody response against furin and to provide further evidence on the safety of the furin-targeting vaccination, we conducted another immunization experiment with both unconjugated and noro-VLP-conjugated P domain and used a prolonged vaccination regime and included an Al(OH)_3_ adjuvant. While additional rounds of immunizations can improve the homogeneity of the B cell response, including the IgG titers,^53^ Al(OH)_3_ is a well-known inducer of antibody production *via* Th2 type T cells with a substantial impact to the overall antibody levels.^54^ We also switched to s.c. injection, which has been reported to boost antibody responses over i.m. injection.^55^ Surprisingly, after two immunizations the anti-furin antibody response was lower in the adjuvanted noro-VLP-P domain group (second immunization experiment) than in the unadjuvanted one (first immunization experiment) and was detectable only after the third immunization. Mechanistically this could be accounted for the lower conjugation efficiency of the second experiment, but the heterogeneity of the B cell response observed in both experiments would suggest that other unidentified factors are additionally involved in the phenomena. The anti-furin antibody response benefitted slightly from the fourth immunization, but for noro-VLP and SpyCatcher, the three-immunization regime seemed optimal for strongest antibody responses. The groups that received unconjugated SpyCatcher-P domain, produced the strongest anti-furin responses in the adjuvanted immunization (Fig. 4B). We have earlier suggested that at least with a conventional adjuvant, SpyCatcher can function as an immune activator,^48^ and the current study strengthens this hypothesis. Previous studies have also implicated that immunity against vaccine platform components could negatively impact the efficacy of vaccination. For example, pre-existing antibodies against adenoviruses can dampen functionality of adenoviral therapies or even cause IgG-mediated toxicity.^56^ Indeed, additional studies are required to understand whether the immunostimulatory activity of SpyCatcher is preserved and to verify that toxicity is not observed upon repeated vaccinations.

We have previously demonstrated that conditional KO of *Furin* in *Cd4-Cre-Furin*^flox/flox^ mice causes T cell expansion and excessive activation of CD4^+^ (helper T cell, Th) and CD8^+^ T cells (cytotoxic T cell, Tc), characterized by increased cell frequencies and excessive IFN-γ and TNF production, respectively.^37,38^ In addition, myeloid cell specific KO of *Furin* in *LysM-Cre-Furin*^flox/flox^ mice leads to increased levels of serum IL-1b in steady state and IL-6 and TNF upon LPS challenge.^36^ Consequently, we assessed the lymphocyte frequencies and the activation status of splenic T cells (production of IFN-γ and TNF) from the P-domain vaccinated mice of the second immunization protocol as well as the quantities of serum IFN-γ, IL-10, IL-12p70, IL-1β, IL-2, IL-4, IL-5, IL-6, KC/gro and TNF. Here, neither flow cytometric analysis nor the multiplex cytokine/chemokine quantification revealed any vaccination-induced inflammation or unspecific T cell activation, providing further proof on the safety of furin vaccination.

## Conclusions

In summary, we used the SpyCatcher/SpyTag system to conjugate two peptides from PCSK9 and a peptide and protein domain from furin with noro-VLPs to create VLP-based vaccine candidates. Without an adjuvant, the highest serum IgG titers were obtained with the PEED peptide from PCSK9 and the furin P domain vaccine candidates. Interestingly, a four-dose immunization using an Al(OH)_3_ adjuvant generated only slightly higher titers against furin but with increased variance within groups. Our data also suggest T cell memory responses against noro-VLP and furin P domain. Importantly, no apparent adverse effects were observed with vaccinations against furin. Future studies should investigate the biological significance of the observed anti-PCSK responses.

## Data availability

The data supporting this article have been included as Additional Information. Other datasets used and/or analyzed during this study are available from the corresponding authors upon reasonable request. The plasmids and full sequences used to create the recombinant protein antigens in this article are available from Addgene (https://www.addgene.org).

## Author contributions

MP and VH conceived the original idea for the project and developed the idea together into the final research plan with VL, MO and MMH. VL produced the vaccines and performed the immunizations and experiments with MO in the supervision of MMH, VH and MP. FMC performed the splenocyte stimulation experiments. SG helped with the animal experiments. VL and MO analyzed and interpreted the results with contributions from VH, MP and MMH. VL and MO drafted the manuscript and figures. All authors discussed the results and commented on the manuscript to help shape its final version. All authors read and approved the final manuscript.

## Conflicts of interest

The authors declare that they have no conflicts of interest with the contents of this article.

## Supplementary Material

NA-OLF-D4NA00483C-s001

NA-OLF-D4NA00483C-s002
