# Supplementary material for: Experimental VLP vaccine displaying a furin antigen elicits production of autoantibodies and is well tolerated in mice
Source: Nanoscale Adv. 2024 Oct 9;6(24):6239–52. doi: 10.1039/d4na00483c (PMC11485048; doi:10.1039/d4na00483c)
Supplement: NA-006-D4NA00483C-s001 [file NA-006-D4NA00483C-s001.pdf]

Supplementary Information (SI) for Nanoscale Advances.
This journal is © The Royal Society of Chemistry 2024

Supplementary information

Experimental VLP vaccine displaying a furin antigen elicits production of autoantibodies and is well tolerated in mice

Vili Lampinen[1*], Markus J. T. Ojanen[1*], Fernanda Muñoz Caro[1], **Stina Gröhn**[1], Minna M. Hankaniemi[1], Marko Pesu[1,2], Vesa P. Hytönen[1,2#]

[1]Faculty of Medicine and Health Technology, Tampere University, Tampere, Finland

[2]Fimlab Laboratories Ltd, Tampere, Finland

[*]Equal contribution

[&]Current address: Finnish Medicines Agency, P.O. Box 55, FI-00034 FIMEA, Finland

[#]Corresponding author: Vesa Hytönen, vesa.hytonen@tuni.fi, Faculty of Medicine and Health Technology, Tampere University, FI-33014 Tampere, Finland

List of supplementary **information**

Supplementary figure 1

Supplementary figure 2

Supplementary figure 3

Supplementary figure 4

Supplementary figure 5

Supplementary figure 6

Supplementary figure 7

Supplementary figure 8

Supplementary figure 9

Supplementary data 1 (separate .xlsx file “Additional file 2”)

A)

SpyTag-noro-VLP capsid protein 1 (557 amino acids, 60.9 kDa):

MKMASSDANPSDGSAANLVPEVNNEVMALEPVVGAAIAAPVAGQQNVIDPWIRNNFVQAPGGEFTVSPRNA
PGEILWSAPLGPDLNPYLSHLARMYNGYAGGFEVQVILAGNAFTAGKVIFAAVPPNFPTEGLSPSQVTMFPHIVV
DVRQLEPVLIPLPDVRNNFYHYNQSNDPTIKLIAMLYTPLRANNAGDDVFTVSCRVLTRPSPDFDFIFLVPPTVESR
TKPFSVPVLTVEEMTNSRFPIPLEKLFTGPSSAFVVQPQNGRCTTDGVLLGTTQLSPVNICTFRGDVTHITGSRNYT
MNLASQNWNDYDPTEEIPAPLGTPDFVGKIQGVLTQTTRTDGSTRGHKATVYTGSADFAPKLGRVQFETDTDRD
FEANQNTKFTPVGVIQDGGTTHRNEPQQWVLPSYSGRNTHNVHLAPAVAPTFPGEQLLFFRSTMPGCSGYPN
MDLDCLLPQEWVQYFYQEAAPAQSDVALLRFVNPDTGRVLFECKLHKSGYVTVAHTGQHDLVIPPNGYFRFDS
WVNQFYTLAPMGNGTGRRRAVTSGGAHIVMVDAYKPTK

B)

SpyCatcher-PEED (122 amino acids, 13.7 kDa):

MHHHHHHDYDIPTTENLYFQGSGDSATHIKFSKRDEDGKELAGATMELRDSSGKTISTWISDGQVKDFYLYPGKY
TFVETAAPDGYEVATAITFTVNEQGQVTVNGLEPEEDGTRFHRQASK

SpyCatcher-DIIG (120 amino acids, 13.2 kDa):
MHHHHHHDYDIPTTENLYFQGSGDSATHIKFSKRDEDGKELAGATMELRDSSGKTISTWISDGQVKDFYLYPGKY
TFVETAAPDGYEVATAITFTVNEQGQVTVNGLEDIIGASSDCSTC

SpyCatcher-SWG (122 amino acids, 13.5 kDa):
MHHHHHHDYDIPTTENLYFQGSGDSATHIKFSKRDEDGKELAGATMELRDSSGKTISTWISDGQVKDFYLYPGKY
TFVETAAPDGYEVATAITFTVNEQGQVTVNGLESWGPEDDGKTVDGP

SpyCatcher-P domain (241 amino acids, 27.0 kDa):
MHHHHHHDYDIPTTENLYFQGSGDSATHIKFSKRDEDGKELAGATMELRDSSGKTISTWISDGQVKDFYLYPGKY
TFVETAAPDGYEVATAITFTVNEQGQVTVNGLEVAPQRKCIVEILVEPKDIGKRLEVRKAVTACLGEPNHITRLEHV
QARLTLSYNRRGDLAIHLISPMGTRSTLLAARPHDYSADGFNDWAFMTTHSWDEDPAGEWVLEIENTSEANNY
GTLTKFTLVLYGTAPE

Supplementary figure 1. Primary amino acid sequences of the vaccine components used for immunizations. A) A noro-VLP capsid protein 1 (black) with a C-terminal SpyTag (light blue). Linker sequence between the viral protein and SpyTag is marked with grey. B) The SpyCatcher-conjugated (dark blue) target regions (black) with an N-terminal purification tag (his-tag, red) and TEV-endonuclease site (orange). Linker sequences are marked with grey. In A and B, the isopeptide bond forming amino acids of both SpyTag- and SpyCatcher-moiety are highlighted with bolded and underlined letters.

A)

```
PCSK1        VGGIRMLDGI-VTDAIEASSIG---FNP---GHVDIYSASWGPNDDGKTVE-GPGRLAQK    286
PCSK2        VAGIRMLDQPFMTDIIEASSIS---HMP---QLIDIYSASWGPTDNGKTVD-GPRELTLQ    287
PCSK3/FURIN  IGGVRMLDGE-VTDAVEARSLG---LNP---NHIHIYSASWGPEDDGKTVD-GPARLAEE    272
PCSK4        IGGVRMLDGT-ITDVIEAQSLS---LQP---QHIHIYSASWGPEDDGRTVD-GPGILTRE    277
PCSK5        IGGVRMLDGD-VTDMVEAKSVS---FNP---QHVHIYSASWGPDDDGKTVD-GPAPLTRQ    290
PCSK6        IGGIRMLDGD-VTDVVEAKSLG---IRP---NYIDIYSASWGPDDDGKTVD-GPGRLAKQ    324
PCSK7        IAGIRVLDGP-LTDSMEAVAFN---KHY---QINDIYSCSWGPDDDGKTVD-GPHQLGKA    306
             :.*:*:**   :** :** :..            .***.**** *:*:**: **  *
PCSK8/MBTP1  LHIFRVFTNN----QVSYTSWFLDAFNYAILKKIDVLNLSI----------GGPDFM-DH    316
PCSK9        MRSLRVLNCQ-GKGTVSGTLIG---LEF---IRKSQLVQPVGPLVVLLPLAGGYSRVLNA    299
```

B)

```
Human  445  VAPQRKCIIDILTEPKDIGKRLEVRKTVTACLGEPNHITRLEHAQARLTLSYNRRGDLAI  503
            VAPQRKCI++IL EPKDIGKRLEVRK VTACLGEPNHITRLEH QARLTLSYNRRGDLAI
Mouse  445  VAPQRKCIVEILVEPKDIGKRLEVRKAVTACLGEPNHITRLEHVQARLTLSYNRRGDLAI  503

Human  504  HLVSPMGTRSTLLAARPHDYSADGFNDWAFMTTHSWDEDPSGEWVLEIENTSEANNYGTL  563
            HL+SPMGTRSTLLAARPHDYSADGFNDWAFMTTHSWDEDP+GEWVLEIENTSEANNYGTL
Mouse  504  HLISPMGTRSTLLAARPHDYSADGFNDWAFMTTHSWDEDPAGEWVLEIENTSEANNYGTL  563

Human  564  TKFTLVLYGTAPE  576
            TKFTLVLYGTAPE
Mouse  564  TKFTLVLYGTAPE  576
```

Supplementary figure 2. A) A section of a multiple sequence alignment of the conventional PCSK family protein sequences from human. The SWG peptide antigen used in this study is highlighted in purple. The human sequences of unconventional PCSK proteins PCSK8/MBTP1 and PCSK9 are shown aligned below the conventional proteins. B) An alignment between the protein sequences of furin P domain of *Homo sapiens* and *Mus musculus*.

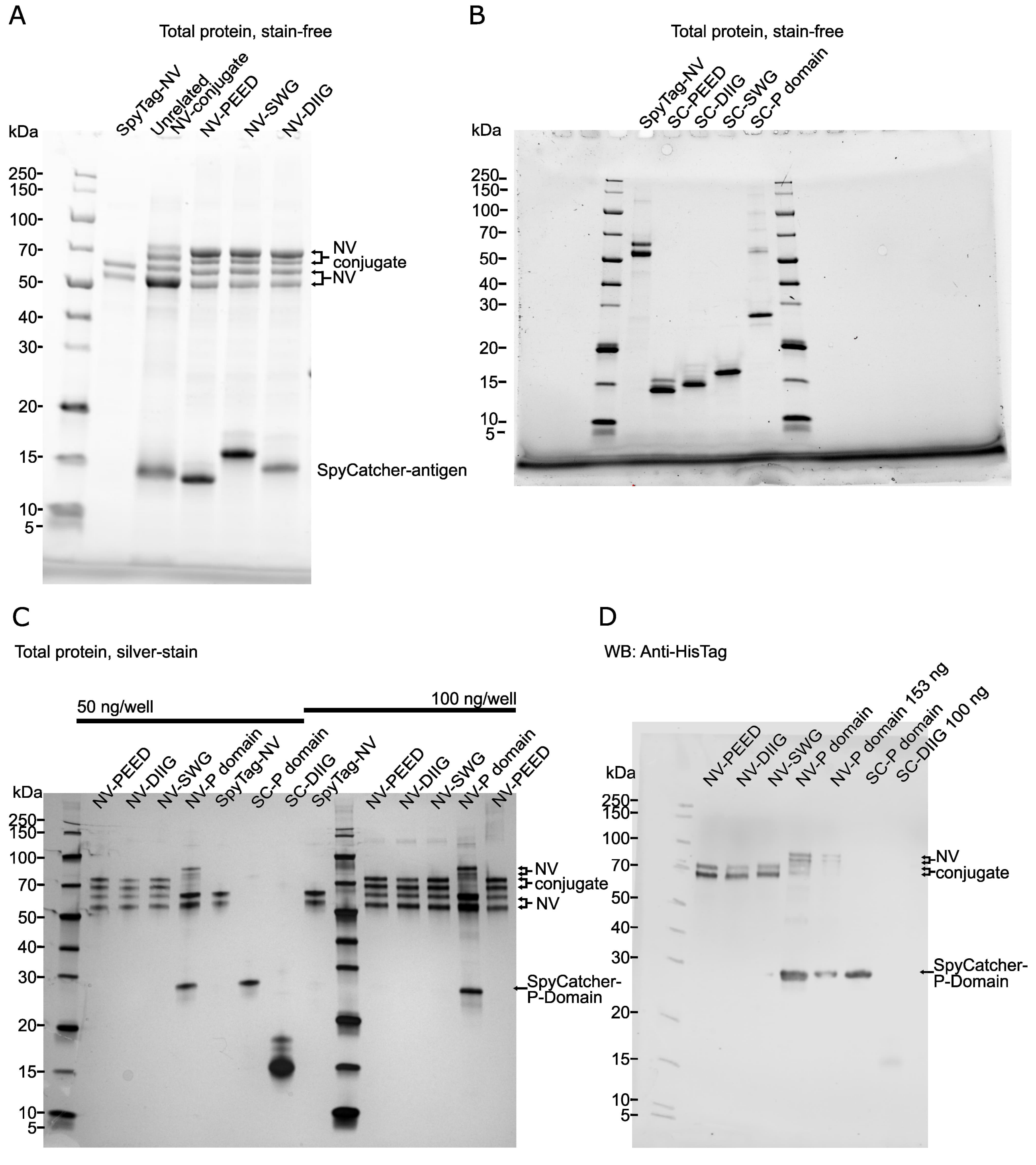

Supplementary figure 3. Characterization of noro-VLP (NV) based vaccine candidates against PCSK9 and furin. A) Stain-free SDS-PAGE gel showing the purified SpyCatcher PCSK antigens combined with SpyTag-NV before the removal of unreacted SpyCatcher-peptides. B) Uncropped gel from Figure 1C. C) Uncropped gel from Figure 1D. D) For Western blotting, we transferred the proteins onto nitrocellulose membrane using Trans-blot Turbo (Bio-Rad). The SpyCatcher-PCSK fusion proteins were identified based on the binding of an HisTag antibody (1:10 000, Thermo Fisher Scientific,

#ma1-21315) and IRDye 800CW goat anti-mouse IgG secondary antibody (1:20 000, LI-COR Biosciences, USA, #926–32210) with the Odyssey CLx instrument (LI-COR Biosciences, USA). All wells have between 500 and 2500 ng protein, unless otherwise mentioned. The molecular weight ladder is marked in the blot with a fluorescent pen.

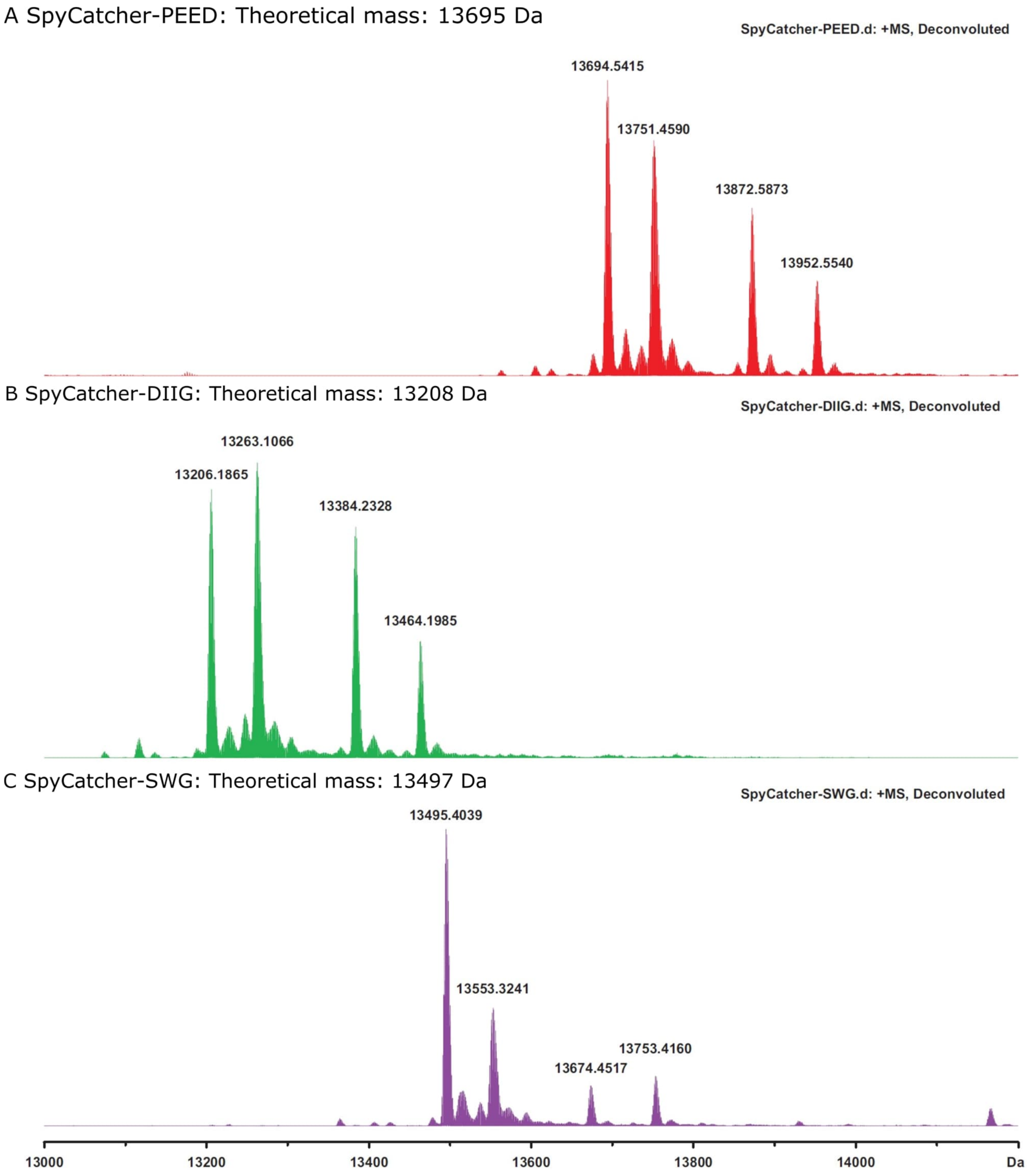

Supplementary **fi**gure 4. Deconvoluted masses of SpyCatcher-fused peptide antigens PEED (A) and DIIG (B) from PCSK9 and SWG (C) from furin were evaluated by matrix-assisted laser desorption/ionization mass spectrometry. The lowest measured masses correspond to the theoretical masses of the SpyCatcher-PEED, -DIIG and -SWG fusion proteins (13695, 13208 and 13497 Da, respectively). Note that the initiating methionine residues have been included in theoretical values.

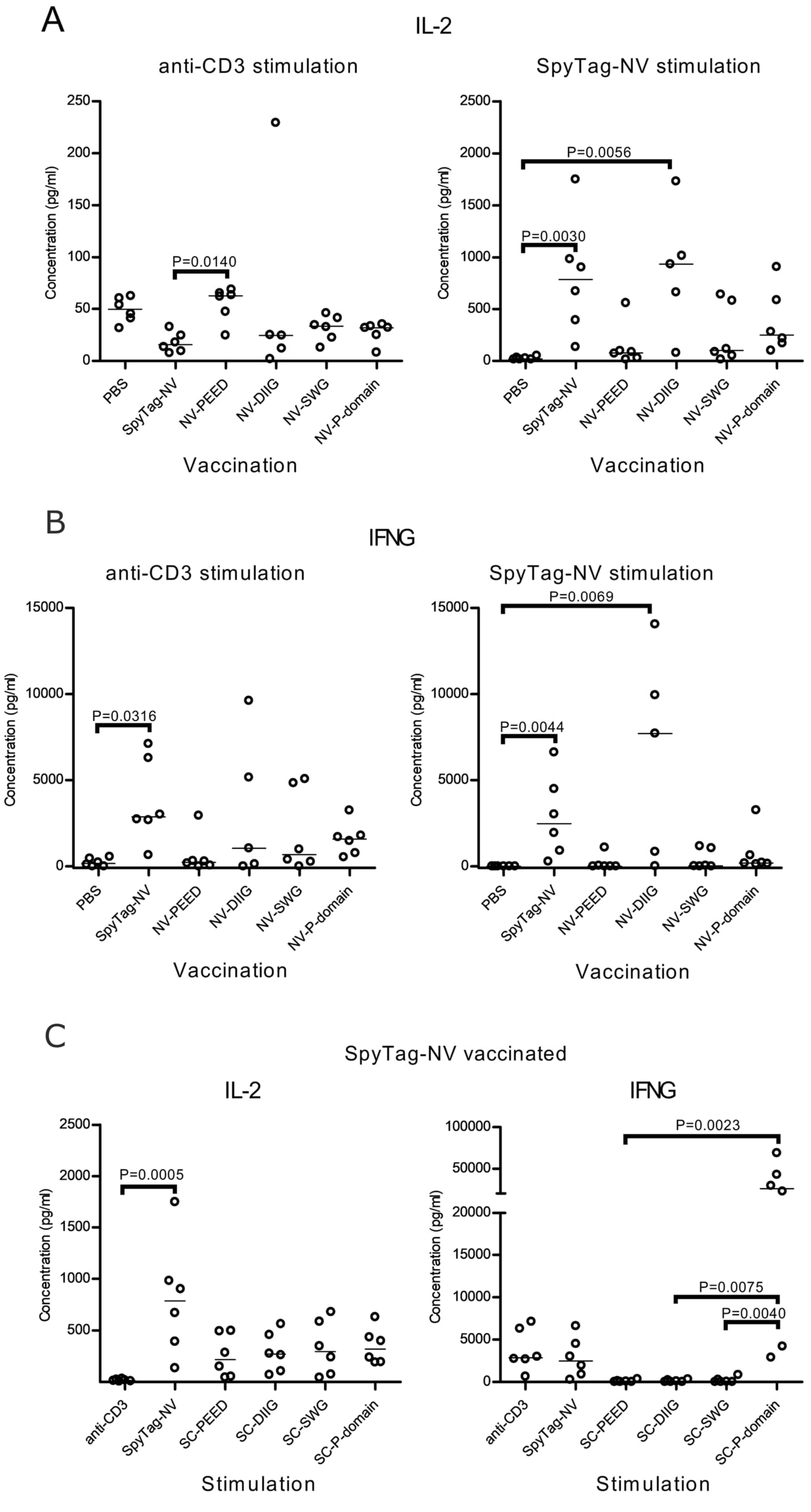
A
IL-2
anti-CD3 stimulation
SpyTag-NV stimulation
Concentration (pg/ml)
P=0.0140
P=0.0056
P=0.0030
PBS
SpyTag-NV
NV-PEED
NV-DIIG
NV-SWG
NV-P-domain
Vaccination
B
IFNG
anti-CD3 stimulation
SpyTag-NV stimulation
Concentration (pg/ml)
P=0.0316
P=0.0069
P=0.0044
PBS
SpyTag-NV
NV-PEED
NV-DIIG
NV-SWG
NV-P-domain
Vaccination
C
SpyTag-NV vaccinated
IL-2
IFNG
Concentration (pg/ml)
P=0.0005
P=0.0023
P=0.0075
P=0.0040
anti-CD3
SpyTag-NV
SC-PEED
SC-DIIG
SC-SWG
SC-P-domain
Stimulation

Supplementary figure 5. T cell recall responses were evaluated from the culture media of *in vitro* stimulated splenocytes. Splenocytes were isolated from 13-week-old PBS, SpyTag-noro-VLP (NV) or antigen-conjugated noro-VLP immunized BALB/c mice (n=5-6, all females), and the cells were stimulated *in vitro* for 3 days at 37°C. The culture media was collected at 3 dps and the IL-2 and IFN-γ (IFNG) concentrations quantified using ELISA. Samples were stimulated using anti-CD3 antibody or SpyTag-NV for the evaluation of unspecific and SpyTag-NV mediated IL-2 (A) and IFN-γ (B) recall responses, respectively. In C), the antigen-specificity of the IL-2 and IFN-γ response is depicted for SpyTag-NV vaccinated mice. Scatter dot plot with median is shown for each group. Nonparametric Kruskal-Wallis one way ANOVA followed by Dunnet/Dunn's test was for the statistical evaluation of differences.

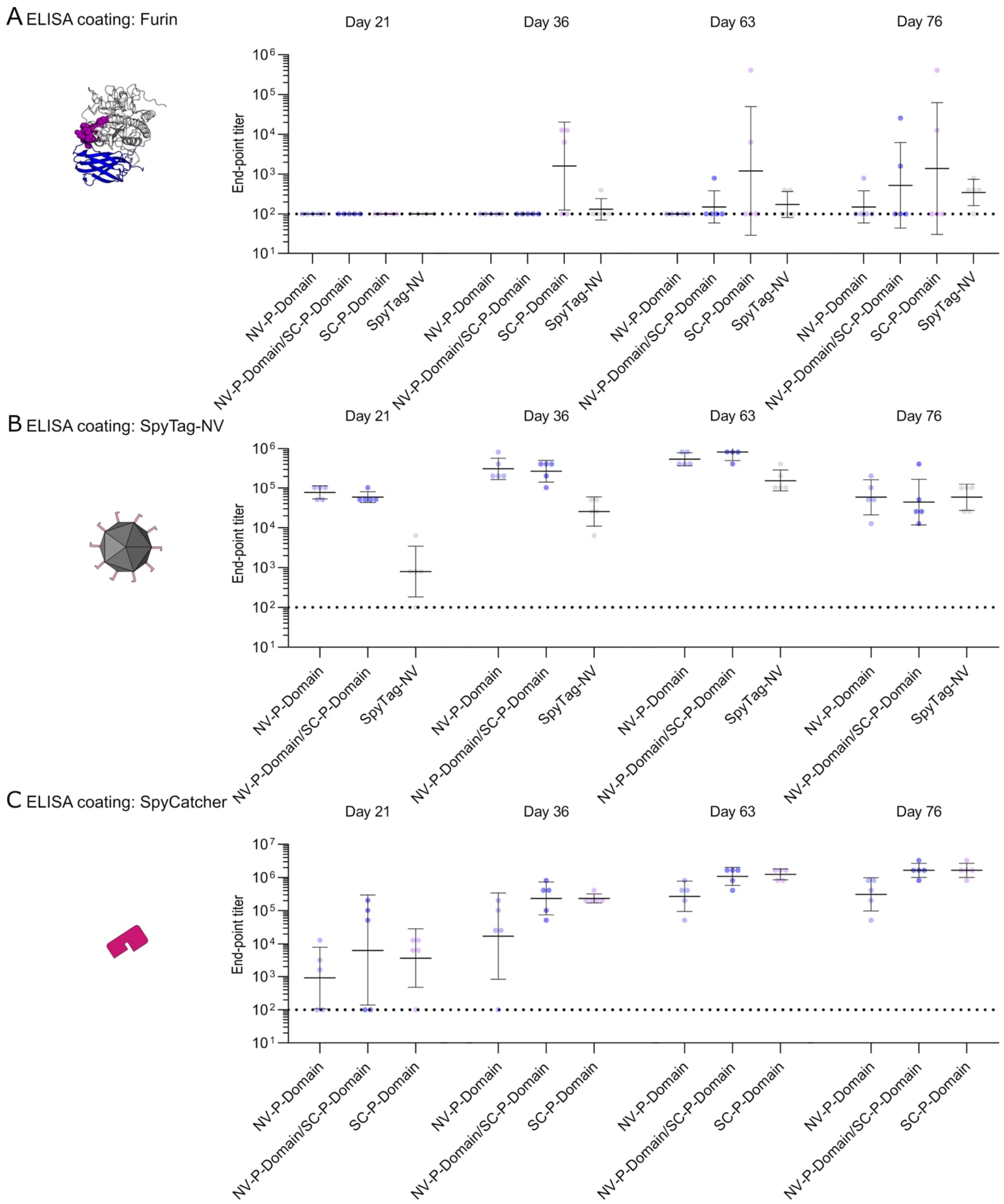

Supplementary figure 6. Adjuvanted vaccination with noro-VLP and SpyCatcher-fused furin P domain yields high anti-furin titers in individual mice. BALB/c mice (n=5, all females) were injected four times s.c. with alum adjuvanted P domain vaccine candidates, SpyTag-noro-VLP (NV), or TBS at days 0, 21, 42, and 63. Tail vein blood samples were collected at days 21, 36, 63, and on day 76 the animals were euthanized, and blood and spleen collected for analysis. Antibody titers were measured using ELISA. Plates were coated with the protein and the reciprocals of IgG antibody titers against recombinant furin (A), SpyTag-noro-VLP (B), and SpyCatcher (C) are shown. The end-point

titer is the reciprocal of the lowest serum dilution that gives a significantly higher absorbance as compared to negative control (TBS) mouse group serum that is diluted 1:400. Mean titers ± standard deviation are depicted. Each dot represents a serum sample from a single mouse. Undetectable antibody levels were denoted with the reciprocal titer 100 (vertical dashed line).

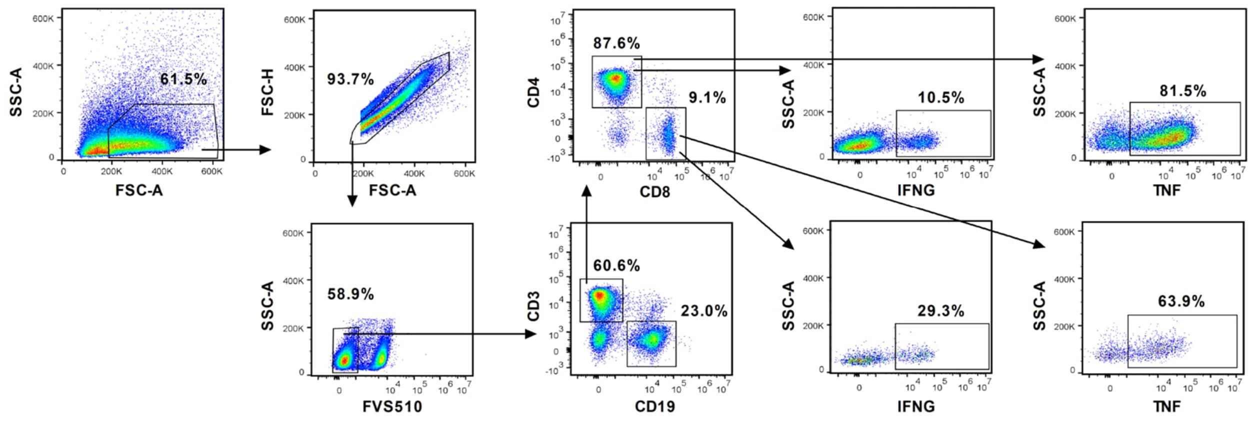

Supplementary figure 7. A representative gating strategy used for the flow cytometric analysis. Note that IFN-γ (IFNG) and TNF were analyzed separately from the samples. SSC = Side scatter, FSC = Forward scatter.

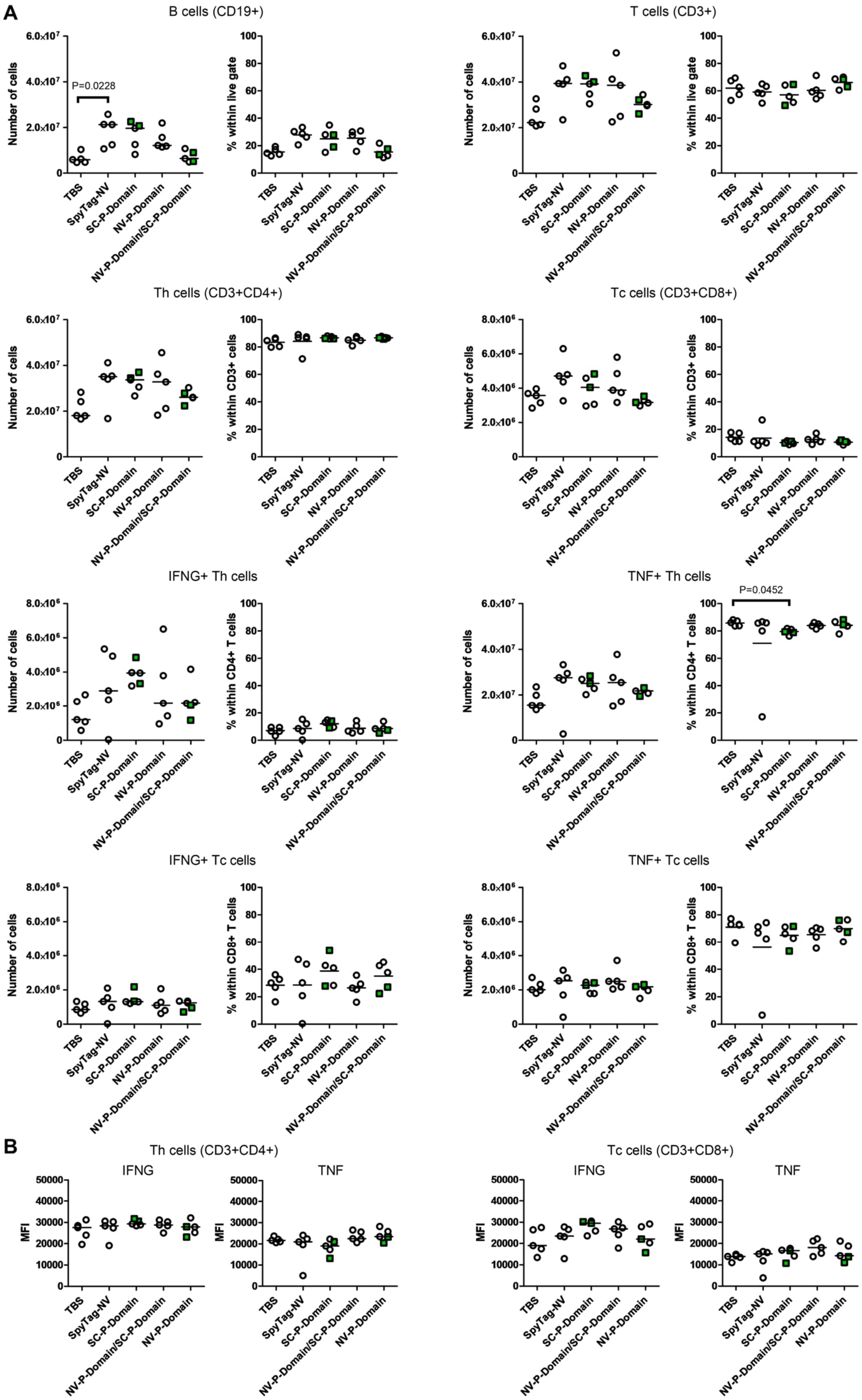
A
B cells (CD19+)
T cells (CD3+)
Th cells (CD3+CD4+)
Tc cells (CD3+CD8+)
IFNG+ Th cells
TNF+ Th cells
IFNG+ Tc cells
TNF+ Tc cells
P=0.0228
P=0.0452
Number of cells
% within live gate
% within CD3+ cells
% within CD4+ T cells
% within CD8+ T cells
TBS
SpyTag-NV
SC-P-Domain
NV-P-Domain
NV-P-Domain/SC-P-Domain
B
Th cells (CD3+CD4+)
Tc cells (CD3+CD8+)
IFNG
TNF
MFI

Supplementary figure 8. Complete dataset of lymphocyte populations and the IL-2 and IFN-γ producing T cells from the adjuvanted vaccination experiment. Splenocytes were isolated from 4-month-old BALB/c mice (n=5, all females) immunized with TBS, unconjugated SpyTag-noro-VLP (NV) or NV-furin-P domain, and the cells were analyzed using flow cytometry. A) The total cell numbers and the relative frequencies of the splenic lymphocyte populations were determined for each mouse. Target populations were identified based on their expression of CD19, CD3, CD4, CD8, IFN-γ (IFNG) and TNF. B) The mean fluorescence intensity (MFI) of IFN-γ+ and TNF+ helper (Th) and cytotoxic (Tc) T cells. Nonparametric Kruskal-Wallis one way ANOVA followed by Dunnet/Dunn's test was used for the statistical evaluation of differences.

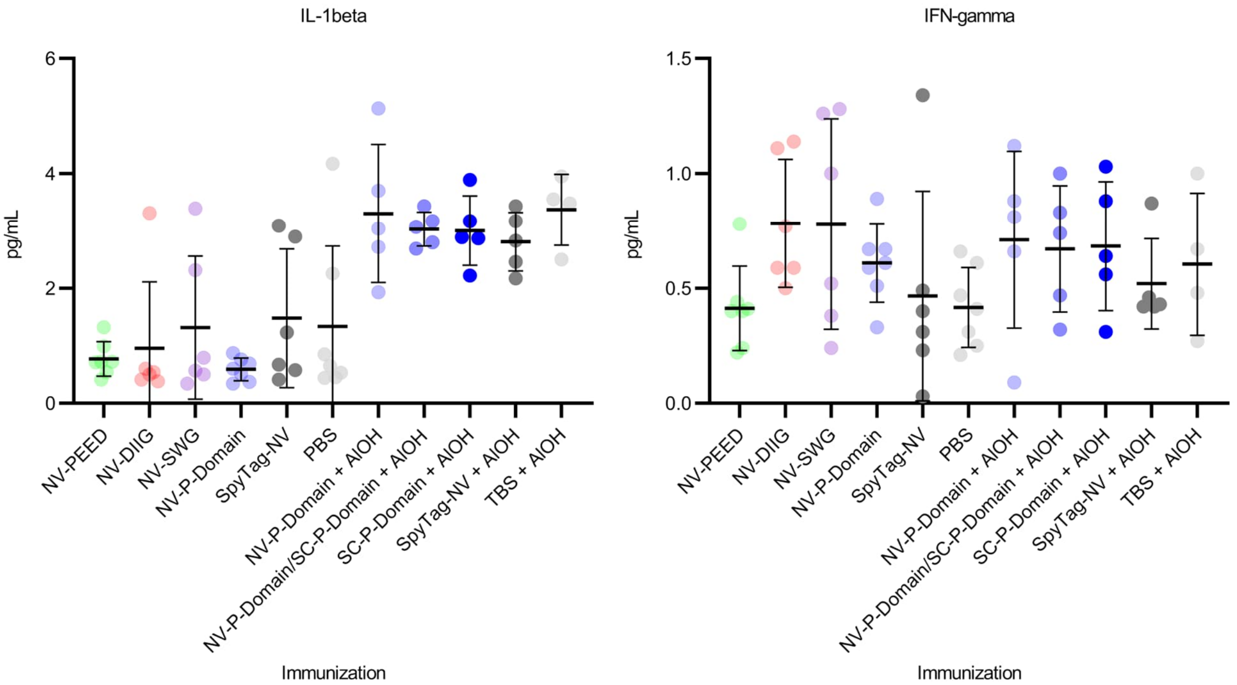

Supplementary figure 9. Mouse serum IL-1β and IFN-γ concentrations were measured with the Meso Scale Discovery multiplex assay kit. The first six vaccination groups on the left-hand side of the graphs are from the first immunization experiment and the last five are the adjuvanted groups from the second immunization experiment. Only differences between groups were observed in IL-1β levels, while IFN-γ serves as a representative example of the rest of the data. All data is available in Supplementary data 1. More specific information about the groups and doses is listed in Table 1.

Supplementary data 1 **in Additional file 2**. Mouse serum IFN-γ, IL-10, IL-12p70, IL-1β, IL-2, IL-4, IL-5, IL-6, KC/gro, and TNF concentrations were measured with the Meso Scale Discovery multiplex assay kit. The sample origins, mouse groups and their abbreviations are shown in tab "Index". Mice encoded with "A" are from the first, unadjuvanted immunization experiment and the ones encoded "B" are from the second immunization experiment. The rest of the data sheets show the raw data from the measurement for each mouse, with the calculated concentration of the cytokine specified in the sheet title. Concentration calculation is based on the raw signal and a standard curve, according to the kit's instructions. 10 out of 64 serum samples were measured as duplicates, and for these, the standard deviation of concentration and standard deviation divided by mean concentration is shown in the tables. Concentrations below the fit curve range are calculated in the table, but calculated negative concentrations are shown as "NaN", instead. More specific information about the groups and doses is listed in Table 1.
