# Supplementary material for: Experimental VLP vaccine displaying a furin antigen elicits production of autoantibodies and is well tolerated in mice
Source: Nanoscale Adv. 2024 Oct 9;6(24):6239–52. doi: 10.1039/d4na00483c (PMC11485048; doi:10.1039/d4na00483c)
Supplement: NA-006-D4NA00483C-s002 [file NA-006-D4NA00483C-s002.pdf]

Supplementary Information (SI) for Nanoscale Advances.
This journal is © The Royal Society of Chemistry 2024

Index

1st immunization experiment=A

Group number	Mouse code	Vaccination group	Duplicate
1	A1.1	PBS	No
1	A1.2	PBS	No
1	A1.3	PBS	No
1	A1.30	PBS	No
1	A1.4	PBS	No
1	A1.5	PBS	No
1	A1.6	PBS	No
2	A2.10	PEED-noro-VLP-SpyCatcher (PCSK9)	No
2	A2.11	PEED-noro-VLP-SpyCatcher (PCSK9)	No
2	A2.12	PEED-noro-VLP-SpyCatcher (PCSK9)	No
2	A2.29	PEED-noro-VLP-SpyCatcher (PCSK9)	Yes
2	A2.7	PEED-noro-VLP-SpyCatcher (PCSK9)	No
2	A2.8	PEED-noro-VLP-SpyCatcher (PCSK9)	No
2	A2.9	PEED-noro-VLP-SpyCatcher (PCSK9)	No
3	A3.13	SWG-noro-VLP-SpyCatcher(furin)	No
3	A3.14	SWG-noro-VLP-SpyCatcher(furin)	No
3	A3.15	SWG-noro-VLP-SpyCatcher(furin)	No
3	A3.16	SWG-noro-VLP-SpyCatcher(furin)	No
3	A3.17	SWG-noro-VLP-SpyCatcher(furin)	No
3	A3.18	SWG-noro-VLP-SpyCatcher(furin)	Yes
4	A4.19	SpyTag-noro-VLP	No
4	A4.20	SpyTag-noro-VLP	No
4	A4.21	SpyTag-noro-VLP	No
4	A4.22	SpyTag-noro-VLP	No
4	A4.23	SpyTag-noro-VLP	No
4	A4.24	SpyTag-noro-VLP	No
4	A4.28	SpyTag-noro-VLP	Yes
5	A5.25	DIIG-noro-VLP-SpyCatcher (PCSK9)	No
5	A5.31	DIIG-noro-VLP-SpyCatcher (PCSK9)	Yes
5	A5.33	DIIG-noro-VLP-SpyCatcher (PCSK9)	No
5	A5.34	DIIG-noro-VLP-SpyCatcher (PCSK9)	No
5	A5.35	DIIG-noro-VLP-SpyCatcher (PCSK9)	No
5	A5.36	DIIG-noro-VLP-SpyCatcher (PCSK9)	No
6	A6.27	Pdomain-noro-VLP-SpyCatcher (furin)	No
6	A6.37	Pdomain-noro-VLP-SpyCatcher (furin)	No
6	A6.38	Pdomain-noro-VLP-SpyCatcher (furin)	No
6	A6.39	Pdomain-noro-VLP-SpyCatcher (furin)	No
6	A6.40	Pdomain-noro-VLP-SpyCatcher (furin)	No
6	A6.41	Pdomain-noro-VLP-SpyCatcher (furin)	Yes
6	A6.42	Pdomain-noro-VLP-SpyCatcher (furin)	No

2nd experiment=B

Group number	Mouse code	Vaccination group	Duplicate
1	B1.1	noro+SC-Pdom+AlOH	No
1	B1.10	noro+SC-Pdom+AlOH	No
1	B1.11	noro+SC-Pdom+AlOH	Yes
1	B1.3	noro+SC-Pdom+AlOH	No
1	B1.30	noro+SC-Pdom+AlOH	No
2	B2.1	noro+SC-Pdom/SC-Pdomain+AlOH	No
2	B2.10	noro+SC-Pdom/SC-Pdomain+AlOH	No
2	B2.11	noro+SC-Pdom/SC-Pdomain+AlOH	Yes
2	B2.3	noro+SC-Pdom/SC-Pdomain+AlOH	No
2	B2.30	noro+SC-Pdom/SC-Pdomain+AlOH	No
3	B3.1	SpyTag-noro-VLP+AlOH	No
3	B3.10	SpyTag-noro-VLP+AlOH	No
3	B3.11	SpyTag-noro-VLP+AlOH	Yes
3	B3.3	SpyTag-noro-VLP+AlOH	No
3	B3.30	SpyTag-noro-VLP+AlOH	No
4	B4.1	SpyCatcher-Pdomain+AlOH	No
4	B4.10	SpyCatcher-Pdomain+AlOH	No
4	B4.11	SpyCatcher-Pdomain+AlOH	Yes
4	B4.3	SpyCatcher-Pdomain+AlOH	No
4	B4.30	SpyCatcher-Pdomain+AlOH	No
5	B5.1	TBS+AlOH	No
5	B5.10	TBS+AlOH	No
5	B5.11	TBS+AlOH	Yes
5	B5.3	TBS+AlOH	No
5	B5.30	TBS+AlOH	No

IFNgamma

Group	Mouse code	Raw signal	Calc. Concent	STD DEV concentration	STD DEV concentration/mean concentration (%)	Detection Range
PBS	A1.1	320	0.61			Below Detection Range
PBS	A1.2	260	0.31			Below Fit Curve Range
PBS	A1.3	220	0.21			Below Fit Curve Range
PBS	A1.30	279	0.41			Below Fit Curve Range
PBS	A1.4	292	0.47			Below Detection Range
PBS	A1.5	330	0.66			In Detection Range
PBS	A1.6	247	0.25			Below Fit Curve Range
PEED-noro-VLP-SpyCatcher (PCSK9)	A2.10	286	0.44			Below Detection Range
PEED-noro-VLP-SpyCatcher (PCSK9)	A2.11	278	0.40			Below Fit Curve Range
PEED-noro-VLP-SpyCatcher (PCSK9)	A2.12	243	0.22			Below Fit Curve Range
PEED-noro-VLP-SpyCatcher (PCSK9)	A2.29	278	0.40	0.01	2.5	Below Fit Curve Range
PEED-noro-VLP-SpyCatcher (PCSK9)	A2.7	245	0.24			Below Fit Curve Range
PEED-noro-VLP-SpyCatcher (PCSK9)	A2.8	355	0.78			In Detection Range
PEED-noro-VLP-SpyCatcher (PCSK9)	A2.9	279	0.41			Below Fit Curve Range
SWG-noro-VLP-SpyCatcher(furin)	A3.13	245	0.24			Below Fit Curve Range
SWG-noro-VLP-SpyCatcher(furin)	A3.14	459	1.26			In Detection Range
SWG-noro-VLP-SpyCatcher(furin)	A3.15	302	0.52			Below Detection Range
SWG-noro-VLP-SpyCatcher(furin)	A3.16	403	1.00			In Detection Range
SWG-noro-VLP-SpyCatcher(furin)	A3.17	463	1.28			In Detection Range
SWG-noro-VLP-SpyCatcher(furin)	A3.18	274	0.38	0.03	6.5	Below Fit Curve Range
SpyTag-noro-VLP	A4.19	259	0.31			Below Fit Curve Range
SpyTag-noro-VLP	A4.20	278	0.40			Below Fit Curve Range
SpyTag-noro-VLP	A4.21	244	0.23			Below Fit Curve Range
SpyTag-noro-VLP	A4.22	168	NaN			Below Fit Curve Range
SpyTag-noro-VLP	A4.23	208	0.03			Below Fit Curve Range
SpyTag-noro-VLP	A4.24	476	1.34			In Detection Range
SpyTag-noro-VLP	A4.28	295	0.49	0.03	7.1	Below Detection Range
DIIG-noro-VLP-SpyCatcher (PCSK9)	A5.25	427	1.11			In Detection Range
DIIG-noro-VLP-SpyCatcher (PCSK9)	A5.31	433	1.14	0.07	6.5	In Detection Range
DIIG-noro-VLP-SpyCatcher (PCSK9)	A5.33	298	0.50			Below Detection Range
DIIG-noro-VLP-SpyCatcher (PCSK9)	A5.34	354	0.77			In Detection Range
DIIG-noro-VLP-SpyCatcher (PCSK9)	A5.35	317	0.59			Below Detection Range
DIIG-noro-VLP-SpyCatcher (PCSK9)	A5.36	316	0.59			Below Detection Range
Pdomain-noro-VLP-SpyCatcher (furin)	A6.27	380	0.89			In Detection Range
Pdomain-noro-VLP-SpyCatcher (furin)	A6.37	333	0.67			In Detection Range
Pdomain-noro-VLP-SpyCatcher (furin)	A6.38	332	0.67			In Detection Range
Pdomain-noro-VLP-SpyCatcher (furin)	A6.39	320	0.61			Below Detection Range
Pdomain-noro-VLP-SpyCatcher (furin)	A6.40	317	0.59			Below Detection Range
Pdomain-noro-VLP-SpyCatcher (furin)	A6.41	299	0.51	0.01	1.9	Below Detection Range
Pdomain-noro-VLP-SpyCatcher (furin)	A6.42	264	0.33			Below Fit Curve Range
noro+SC-Pdom+AlOH	B1.1	330	0.66			In Detection Range
noro+SC-Pdom+AlOH	B1.10	218	0.09			Below Fit Curve Range
noro+SC-Pdom+AlOH	B1.11	362	0.81	0.00	0.3	In Detection Range
noro+SC-Pdom+AlOH	B1.3	428	1.12			In Detection Range
noro+SC-Pdom+AlOH	B1.30	377	0.88			In Detection Range
noro+SC-Pdom/SC-Pdomain+AlOH	B2.1	292	0.47			Below Detection Range
noro+SC-Pdom/SC-Pdomain+AlOH	B2.10	347	0.74			In Detection Range
noro+SC-Pdom/SC-Pdomain+AlOH	B2.11	366	0.83	0.08	9.4	In Detection Range
noro+SC-Pdom/SC-Pdomain+AlOH	B2.3	261	0.32			Below Fit Curve Range
noro+SC-Pdom/SC-Pdomain+AlOH	B2.30	403	1.00			In Detection Range
SpyTag-noro-VLP+AlOH	B3.1	281	0.42			Below Fit Curve Range
SpyTag-noro-VLP+AlOH	B3.10	283	0.43			Below Fit Curve Range
SpyTag-noro-VLP+AlOH	B3.11	281	0.42	0.04	9.5	Below Fit Curve Range
SpyTag-noro-VLP+AlOH	B3.3	375	0.87			In Detection Range
SpyTag-noro-VLP+AlOH	B3.30	290	0.46			Below Detection Range
SpyCatcher-Pdomain+AlOH	B4.1	409	1.03			In Detection Range
SpyCatcher-Pdomain+AlOH	B4.10	260	0.31			Below Fit Curve Range
SpyCatcher-Pdomain+AlOH	B4.11	378	0.88	0.03	3.7	In Detection Range
SpyCatcher-Pdomain+AlOH	B4.3	326	0.64			In Detection Range
SpyCatcher-Pdomain+AlOH	B4.30	310	0.56			Below Detection Range
TBS+AlOH	B5.1	294	0.48			Below Detection Range
TBS+AlOH	B5.10	252	0.27			Below Fit Curve Range
TBS+AlOH	B5.11	403	1.00	0.02	2.1	In Detection Range
TBS+AlOH	B5.3	332	0.67			In Detection Range

IL-10

Group	Mouse code	Raw signal	Calc. Concent	STD DEV concentration	STD DEV concentration/mean concentration (%)	Detection Range
PBS	A1.1	1034	3.39			In Detection Range
PBS	A1.2	1024	3.31			In Detection Range
PBS	A1.3	614	0.37			Below Fit Curve Range
PBS	A1.30	868	2.16			In Detection Range
PBS	A1.4	855	2.06			In Detection Range
PBS	A1.5	875	2.21			In Detection Range
PBS	A1.6	854	2.06			In Detection Range
PEED-noro-VLP-SpyCatcher (PCSK9)	A2.10	764	1.37			Below Fit Curve Range
PEED-noro-VLP-SpyCatcher (PCSK9)	A2.11	1200	4.59			In Detection Range
PEED-noro-VLP-SpyCatcher (PCSK9)	A2.12	1058	3.56			In Detection Range
PEED-noro-VLP-SpyCatcher (PCSK9)	A2.29	1084	3.75	0.05	1.4	In Detection Range
PEED-noro-VLP-SpyCatcher (PCSK9)	A2.7	633	0.34			Below Fit Curve Range
PEED-noro-VLP-SpyCatcher (PCSK9)	A2.8	1110	3.94			In Detection Range
PEED-noro-VLP-SpyCatcher (PCSK9)	A2.9	925	2.59			In Detection Range
SWG-noro-VLP-SpyCatcher(furin)	A3.13	1004	3.17			In Detection Range
SWG-noro-VLP-SpyCatcher(furin)	A3.14	1179	4.44			In Detection Range
SWG-noro-VLP-SpyCatcher(furin)	A3.15	1187	4.50			In Detection Range
SWG-noro-VLP-SpyCatcher(furin)	A3.16	1481	6.60			In Detection Range
SWG-noro-VLP-SpyCatcher(furin)	A3.17	1028	3.34			In Detection Range
SWG-noro-VLP-SpyCatcher(furin)	A3.18	892	2.34	0.10	4.3	In Detection Range
SpyTag-noro-VLP	A4.19	1011	3.22			In Detection Range
SpyTag-noro-VLP	A4.20	951	2.78			In Detection Range
SpyTag-noro-VLP	A4.21	908	2.46			In Detection Range
SpyTag-noro-VLP	A4.22	513	NaN			Below Fit Curve Range
SpyTag-noro-VLP	A4.23	794	1.60			Below Detection Range
SpyTag-noro-VLP	A4.24	1127	4.07			In Detection Range
SpyTag-noro-VLP	A4.28	1171	4.38	0.06	1.3	In Detection Range
DIIG-noro-VLP-SpyCatcher (PCSK9)	A5.25	1310	5.38			In Detection Range
DIIG-noro-VLP-SpyCatcher (PCSK9)	A5.31	1604	7.46	0.32	4.3	In Detection Range
DIIG-noro-VLP-SpyCatcher (PCSK9)	A5.33	1449	6.37			In Detection Range
DIIG-noro-VLP-SpyCatcher (PCSK9)	A5.34	1065	3.61			In Detection Range
DIIG-noro-VLP-SpyCatcher (PCSK9)	A5.35	1027	3.34			In Detection Range
DIIG-noro-VLP-SpyCatcher (PCSK9)	A5.36	1088	3.78			In Detection Range
Pdomain-noro-VLP-SpyCatcher (furin)	A6.27	1118	4.00			In Detection Range
Pdomain-noro-VLP-SpyCatcher (furin)	A6.37	1193	4.54			In Detection Range
Pdomain-noro-VLP-SpyCatcher (furin)	A6.38	1264	5.05			In Detection Range
Pdomain-noro-VLP-SpyCatcher (furin)	A6.39	1020	3.29			In Detection Range
Pdomain-noro-VLP-SpyCatcher (furin)	A6.40	3075	17.52			In Detection Range
Pdomain-noro-VLP-SpyCatcher (furin)	A6.41	1092	3.81	0.05	1.4	In Detection Range
Pdomain-noro-VLP-SpyCatcher (furin)	A6.42	791	1.58			Below Detection Range
noro+SC-Pdom+AlOH	B1.1	875	2.21			In Detection Range
noro+SC-Pdom+AlOH	B1.10	967	2.90			In Detection Range
noro+SC-Pdom+AlOH	B1.11	978.5	2.98	0.08	2.6	In Detection Range
noro+SC-Pdom+AlOH	B1.3	1365	5.77			In Detection Range
noro+SC-Pdom+AlOH	B1.30	992	3.08			In Detection Range
noro+SC-Pdom/SC-Pdomain+AlOH	B2.1	844	1.98			In Detection Range
noro+SC-Pdom/SC-Pdomain+AlOH	B2.10	905	2.44			In Detection Range
noro+SC-Pdom/SC-Pdomain+AlOH	B2.11	924.5	2.58	0.21	8.2	In Detection Range
noro+SC-Pdom/SC-Pdomain+AlOH	B2.3	858	2.09			In Detection Range
noro+SC-Pdom/SC-Pdomain+AlOH	B2.30	1119	4.01			In Detection Range
SpyTag-noro-VLP+AlOH	B3.1	1033	3.38			In Detection Range
SpyTag-noro-VLP+AlOH	B3.10	862	2.12			In Detection Range
SpyTag-noro-VLP+AlOH	B3.11	1134	4.12	0.11	2.6	In Detection Range
SpyTag-noro-VLP+AlOH	B3.3	1165	4.34			In Detection Range
SpyTag-noro-VLP+AlOH	B3.30	934	2.65			In Detection Range
SpyCatcher-Pdomain+AlOH	B4.1	926	2.59			In Detection Range
SpyCatcher-Pdomain+AlOH	B4.10	937	2.67			In Detection Range
SpyCatcher-Pdomain+AlOH	B4.11	1102	3.88	0.04	0.9	In Detection Range
SpyCatcher-Pdomain+AlOH	B4.3	983	3.01			In Detection Range
SpyCatcher-Pdomain+AlOH	B4.30	897	2.38			In Detection Range
TBS+AlOH	B5.1	964	2.87			In Detection Range
TBS+AlOH	B5.10	1129	4.08			In Detection Range
TBS+AlOH	B5.11	1081	3.73	0.01	0.4	In Detection Range
TBS+AlOH	B5.3	1125	4.05			In Detection Range

IL-12p70

Group	Mouse code	Raw signal	Calc. Concent	STD DEV concentration	STD DEV concentration/mean concentration (%)	Detection Range
PBS	A1.1	361	NaN			Below Fit Curve Range
PBS	A1.2	414	NaN			Below Fit Curve Range
PBS	A1.3	315	NaN			Below Fit Curve Range
PBS	A1.30	325	NaN			Below Fit Curve Range
PBS	A1.4	348	NaN			Below Fit Curve Range
PBS	A1.5	330	NaN			Below Fit Curve Range
PBS	A1.6	330	NaN			Below Fit Curve Range
PEED-noro-VLP-SpyCatcher (PCSK9)	A2.10	370	NaN			Below Fit Curve Range
PEED-noro-VLP-SpyCatcher (PCSK9)	A2.11	351	NaN			Below Fit Curve Range
PEED-noro-VLP-SpyCatcher (PCSK9)	A2.12	327	NaN			Below Fit Curve Range
PEED-noro-VLP-SpyCatcher (PCSK9)	A2.29	352	NaN	NaN	NaN	Below Fit Curve Range
PEED-noro-VLP-SpyCatcher (PCSK9)	A2.7	349	NaN			Below Fit Curve Range
PEED-noro-VLP-SpyCatcher (PCSK9)	A2.8	390	NaN			Below Fit Curve Range
PEED-noro-VLP-SpyCatcher (PCSK9)	A2.9	377	NaN			Below Fit Curve Range
SWG-noro-VLP-SpyCatcher(furin)	A3.13	318	NaN			Below Fit Curve Range
SWG-noro-VLP-SpyCatcher(furin)	A3.14	319	NaN			Below Fit Curve Range
SWG-noro-VLP-SpyCatcher(furin)	A3.15	335	NaN			Below Fit Curve Range
SWG-noro-VLP-SpyCatcher(furin)	A3.16	348	NaN			Below Fit Curve Range
SWG-noro-VLP-SpyCatcher(furin)	A3.17	351	NaN			Below Fit Curve Range
SWG-noro-VLP-SpyCatcher(furin)	A3.18	347	NaN	NaN	NaN	Below Fit Curve Range
SpyTag-noro-VLP	A4.19	351	NaN			Below Fit Curve Range
SpyTag-noro-VLP	A4.20	457	13.35			Below Fit Curve Range
SpyTag-noro-VLP	A4.21	338	NaN			Below Fit Curve Range
SpyTag-noro-VLP	A4.22	351	NaN			Below Fit Curve Range
SpyTag-noro-VLP	A4.23	342	NaN			Below Fit Curve Range
SpyTag-noro-VLP	A4.24	683	59.61			In Detection Range
SpyTag-noro-VLP	A4.28	352	NaN	NaN	NaN	Below Fit Curve Range
DIIG-noro-VLP-SpyCatcher (PCSK9)	A5.25	348	NaN			Below Fit Curve Range
DIIG-noro-VLP-SpyCatcher (PCSK9)	A5.31	371	NaN	NaN	NaN	Below Fit Curve Range
DIIG-noro-VLP-SpyCatcher (PCSK9)	A5.33	365	NaN			Below Fit Curve Range
DIIG-noro-VLP-SpyCatcher (PCSK9)	A5.34	346	NaN			Below Fit Curve Range
DIIG-noro-VLP-SpyCatcher (PCSK9)	A5.35	356	NaN			Below Fit Curve Range
DIIG-noro-VLP-SpyCatcher (PCSK9)	A5.36	377	NaN			Below Fit Curve Range
Pdomain-noro-VLP-SpyCatcher (furin)	A6.27	330	NaN			Below Fit Curve Range
Pdomain-noro-VLP-SpyCatcher (furin)	A6.37	338	NaN			Below Fit Curve Range
Pdomain-noro-VLP-SpyCatcher (furin)	A6.38	354	NaN			Below Fit Curve Range
Pdomain-noro-VLP-SpyCatcher (furin)	A6.39	313	NaN			Below Fit Curve Range
Pdomain-noro-VLP-SpyCatcher (furin)	A6.40	381	NaN			Below Fit Curve Range
Pdomain-noro-VLP-SpyCatcher (furin)	A6.41	353	NaN	NaN	NaN	Below Fit Curve Range
Pdomain-noro-VLP-SpyCatcher (furin)	A6.42	325	NaN			Below Fit Curve Range
noro+SC-Pdom+AlOH	B1.1	358	NaN			Below Fit Curve Range
noro+SC-Pdom+AlOH	B1.10	355	NaN			Below Fit Curve Range
noro+SC-Pdom+AlOH	B1.11	337	NaN	NaN	NaN	Below Fit Curve Range
noro+SC-Pdom+AlOH	B1.3	367	NaN			Below Fit Curve Range
noro+SC-Pdom+AlOH	B1.30	376	NaN			Below Fit Curve Range
noro+SC-Pdom/SC-Pdomain+AlOH	B2.1	343	NaN			Below Fit Curve Range
noro+SC-Pdom/SC-Pdomain+AlOH	B2.10	333	NaN			Below Fit Curve Range
noro+SC-Pdom/SC-Pdomain+AlOH	B2.11	468	16.04	1.64	10.2	Below Detection Range
noro+SC-Pdom/SC-Pdomain+AlOH	B2.3	347	NaN			Below Fit Curve Range
noro+SC-Pdom/SC-Pdomain+AlOH	B2.30	341	NaN			Below Fit Curve Range
SpyTag-noro-VLP+AlOH	B3.1	336	NaN			Below Fit Curve Range
SpyTag-noro-VLP+AlOH	B3.10	336	NaN			Below Fit Curve Range
SpyTag-noro-VLP+AlOH	B3.11	363	NaN	NaN	NaN	Below Fit Curve Range
SpyTag-noro-VLP+AlOH	B3.3	332	NaN			Below Fit Curve Range
SpyTag-noro-VLP+AlOH	B3.30	364	NaN			Below Fit Curve Range
SpyCatcher-Pdomain+AlOH	B4.1	331	NaN			Below Fit Curve Range
SpyCatcher-Pdomain+AlOH	B4.10	303	NaN			Below Fit Curve Range
SpyCatcher-Pdomain+AlOH	B4.11	393	NaN	NaN	NaN	Below Fit Curve Range
SpyCatcher-Pdomain+AlOH	B4.3	311	NaN			Below Fit Curve Range
SpyCatcher-Pdomain+AlOH	B4.30	352	NaN			Below Fit Curve Range
TBS+AlOH	B5.1	318	NaN			Below Fit Curve Range
TBS+AlOH	B5.10	348	NaN			Below Fit Curve Range
TBS+AlOH	B5.11	333	NaN	NaN	NaN	Below Fit Curve Range
TBS+AlOH	B5.3	346	NaN			Below Fit Curve Range

IL-1beta

Group	Mouse code	Raw signal	Calc. Concent	STD DEV concentration	STD DEV concentration/mean concentration (%)	Detection Range
PBS	A1.1	449	0.85			Below Detection Range
PBS	A1.2	346	0.53			Below Fit Curve Range
PBS	A1.3	247	0.45			Below Fit Curve Range
PBS	A1.30	317	0.44			Below Fit Curve Range
PBS	A1.4	1526	4.17			In Detection Range
PBS	A1.5	386	0.65			Below Fit Curve Range
PBS	A1.6	902	2.26			In Detection Range
PEED-noro-VLP-SpyCatcher (PCSK9)	A2.10	408	0.72			Below Fit Curve Range
PEED-noro-VLP-SpyCatcher (PCSK9)	A2.11	406	0.72			Below Fit Curve Range
PEED-noro-VLP-SpyCatcher (PCSK9)	A2.12	601	1.32			In Detection Range
PEED-noro-VLP-SpyCatcher (PCSK9)	A2.29	351	0.54	0.01	2.6	Below Fit Curve Range
PEED-noro-VLP-SpyCatcher (PCSK9)	A2.7	308	0.41			Below Fit Curve Range
PEED-noro-VLP-SpyCatcher (PCSK9)	A2.8	403	0.71			Below Fit Curve Range
PEED-noro-VLP-SpyCatcher (PCSK9)	A2.9	495	0.99			In Detection Range
SWG-noro-VLP-SpyCatcher(furin)	A3.13	336	0.50			Below Fit Curve Range
SWG-noro-VLP-SpyCatcher(furin)	A3.14	285	0.34			Below Fit Curve Range
SWG-noro-VLP-SpyCatcher(furin)	A3.15	430	0.79			Below Detection Range
SWG-noro-VLP-SpyCatcher(furin)	A3.16	1270	3.39			In Detection Range
SWG-noro-VLP-SpyCatcher(furin)	A3.17	923	2.32			In Detection Range
SWG-noro-VLP-SpyCatcher(furin)	A3.18	358	0.57	0.01	1.9	Below Fit Curve Range
SpyTag-noro-VLP	A4.19	362	0.58			Below Fit Curve Range
SpyTag-noro-VLP	A4.20	307	0.41			Below Fit Curve Range
SpyTag-noro-VLP	A4.21	1173	3.09			In Detection Range
SpyTag-noro-VLP	A4.22	146	NaN			Below Fit Curve Range
SpyTag-noro-VLP	A4.23	1110	2.90			In Detection Range
SpyTag-noro-VLP	A4.24	572	1.23			In Detection Range
SpyTag-noro-VLP	A4.28	390	0.67	0.02	2.8	Below Fit Curve Range
DIIG-noro-VLP-SpyCatcher (PCSK9)	A5.25	1246	3.31			In Detection Range
DIIG-noro-VLP-SpyCatcher (PCSK9)	A5.31	337	0.50	0.03	6.6	Below Fit Curve Range
DIIG-noro-VLP-SpyCatcher (PCSK9)	A5.33	369	0.60			Below Fit Curve Range
DIIG-noro-VLP-SpyCatcher (PCSK9)	A5.34	299	0.38			Below Fit Curve Range
DIIG-noro-VLP-SpyCatcher (PCSK9)	A5.35	348	0.54			Below Fit Curve Range
DIIG-noro-VLP-SpyCatcher (PCSK9)	A5.36	309	0.41			Below Fit Curve Range
Pdomain-noro-VLP-SpyCatcher (furin)	A6.27	421	0.76			Below Fit Curve Range
Pdomain-noro-VLP-SpyCatcher (furin)	A6.37	370	0.60			Below Fit Curve Range
Pdomain-noro-VLP-SpyCatcher (furin)	A6.38	286	0.34			Below Fit Curve Range
Pdomain-noro-VLP-SpyCatcher (furin)	A6.39	455	0.87			Below Detection Range
Pdomain-noro-VLP-SpyCatcher (furin)	A6.40	341	0.51			Below Fit Curve Range
Pdomain-noro-VLP-SpyCatcher (furin)	A6.41	296	0.37	0.04	10.1	Below Fit Curve Range
Pdomain-noro-VLP-SpyCatcher (furin)	A6.42	396	0.69			Below Fit Curve Range
noro+SC-Pdom+AlOH	B1.1	798	1.93			In Detection Range
noro+SC-Pdom+AlOH	B1.10	1158	3.04			In Detection Range
noro+SC-Pdom+AlOH	B1.11	1373	3.70	0.16	4.3	In Detection Range
noro+SC-Pdom+AlOH	B1.3	1838	5.13			In Detection Range
noro+SC-Pdom+AlOH	B1.30	1053	2.72			In Detection Range
noro+SC-Pdom/SC-Pdomain+AlOH	B2.1	1080	2.80			In Detection Range
noro+SC-Pdom/SC-Pdomain+AlOH	B2.10	1165	3.07			In Detection Range
noro+SC-Pdom/SC-Pdomain+AlOH	B2.11	1042	2.69	0.10	3.7	In Detection Range
noro+SC-Pdom/SC-Pdomain+AlOH	B2.3	1200	3.17			In Detection Range
noro+SC-Pdom/SC-Pdomain+AlOH	B2.30	1285	3.43			In Detection Range
SpyTag-noro-VLP+AlOH	B3.1	1285	3.43			In Detection Range
SpyTag-noro-VLP+AlOH	B3.10	875	2.17			In Detection Range
SpyTag-noro-VLP+AlOH	B3.11	968	2.46	0.04	1.6	In Detection Range
SpyTag-noro-VLP+AlOH	B3.3	1198	3.17			In Detection Range
SpyTag-noro-VLP+AlOH	B3.30	1089	2.83			In Detection Range
SpyCatcher-Pdomain+AlOH	B4.1	1107	2.89			In Detection Range
SpyCatcher-Pdomain+AlOH	B4.10	1103	2.87			In Detection Range
SpyCatcher-Pdomain+AlOH	B4.11	1199	3.17	0.02	0.5	In Detection Range
SpyCatcher-Pdomain+AlOH	B4.3	890	2.22			In Detection Range
SpyCatcher-Pdomain+AlOH	B4.30	1434	3.89			In Detection Range
TBS+AlOH	B5.1	981	2.50			In Detection Range
TBS+AlOH	B5.10	1301	3.48			In Detection Range
TBS+AlOH	B5.11	1452	3.95	0.34	8.6	In Detection Range
TBS+AlOH	B5.3	1324	3.55			In Detection Range

IL-2

Group	Mouse code	Raw signal	Calc. Concent	STD DEV concentration	STD DEV concentration/mean concentration (%)	Detection Range
PBS	A1.1	294	0.75			Below Fit Curve Range
PBS	A1.2	294	0.75			Below Fit Curve Range
PBS	A1.3	233	NaN			Below Fit Curve Range
PBS	A1.30	280	0.52			Below Fit Curve Range
PBS	A1.4	299	0.83			Below Fit Curve Range
PBS	A1.5	252	0.04			Below Fit Curve Range
PBS	A1.6	361	1.79			In Detection Range
PEED-noro-VLP-SpyCatcher (PCSK9)	A2.10	288	0.65			Below Fit Curve Range
PEED-noro-VLP-SpyCatcher (PCSK9)	A2.11	245	NaN			Below Fit Curve Range
PEED-noro-VLP-SpyCatcher (PCSK9)	A2.12	272	0.39			Below Fit Curve Range
PEED-noro-VLP-SpyCatcher (PCSK9)	A2.29	252	0.30	0.00	0.0	Below Fit Curve Range
PEED-noro-VLP-SpyCatcher (PCSK9)	A2.7	247	NaN			Below Fit Curve Range
PEED-noro-VLP-SpyCatcher (PCSK9)	A2.8	266	0.29			Below Fit Curve Range
PEED-noro-VLP-SpyCatcher (PCSK9)	A2.9	266	0.29			Below Fit Curve Range
SWG-noro-VLP-SpyCatcher(furin)	A3.13	261	0.20			Below Fit Curve Range
SWG-noro-VLP-SpyCatcher(furin)	A3.14	261	0.20			Below Fit Curve Range
SWG-noro-VLP-SpyCatcher(furin)	A3.15	243	NaN			Below Fit Curve Range
SWG-noro-VLP-SpyCatcher(furin)	A3.16	291	0.70			Below Fit Curve Range
SWG-noro-VLP-SpyCatcher(furin)	A3.17	263	0.24			Below Fit Curve Range
SWG-noro-VLP-SpyCatcher(furin)	A3.18	242	0.04	0.00	0.0	Below Fit Curve Range
SpyTag-noro-VLP	A4.19	264	0.25			Below Fit Curve Range
SpyTag-noro-VLP	A4.20	263	0.24			Below Fit Curve Range
SpyTag-noro-VLP	A4.21	243	NaN			Below Fit Curve Range
SpyTag-noro-VLP	A4.22	209	NaN			Below Fit Curve Range
SpyTag-noro-VLP	A4.23	233	NaN			Below Fit Curve Range
SpyTag-noro-VLP	A4.24	442	2.99			In Detection Range
SpyTag-noro-VLP	A4.28	252	0.11	0.00	0.0	Below Fit Curve Range
DIIG-noro-VLP-SpyCatcher (PCSK9)	A5.25	319	1.14			Below Detection Range
DIIG-noro-VLP-SpyCatcher (PCSK9)	A5.31	266	0.29	0.03	11.9	Below Fit Curve Range
DIIG-noro-VLP-SpyCatcher (PCSK9)	A5.33	294	0.75			Below Fit Curve Range
DIIG-noro-VLP-SpyCatcher (PCSK9)	A5.34	289	0.67			Below Fit Curve Range
DIIG-noro-VLP-SpyCatcher (PCSK9)	A5.35	270	0.36			Below Fit Curve Range
DIIG-noro-VLP-SpyCatcher (PCSK9)	A5.36	269	0.34			Below Fit Curve Range
Pdomain-noro-VLP-SpyCatcher (furin)	A6.27	229	NaN			Below Fit Curve Range
Pdomain-noro-VLP-SpyCatcher (furin)	A6.37	270	0.36			Below Fit Curve Range
Pdomain-noro-VLP-SpyCatcher (furin)	A6.38	283	0.57			Below Fit Curve Range
Pdomain-noro-VLP-SpyCatcher (furin)	A6.39	271	0.37			Below Fit Curve Range
Pdomain-noro-VLP-SpyCatcher (furin)	A6.40	257	0.13			Below Fit Curve Range
Pdomain-noro-VLP-SpyCatcher (furin)	A6.41	261	0.20	0.14	71.4	Below Fit Curve Range
Pdomain-noro-VLP-SpyCatcher (furin)	A6.42	258	0.15			Below Fit Curve Range
noro+SC-Pdom+AlOH	B1.1	259	0.17			Below Fit Curve Range
noro+SC-Pdom+AlOH	B1.10	229	NaN			Below Fit Curve Range
noro+SC-Pdom+AlOH	B1.11	238	NaN	NaN	NaN	Below Fit Curve Range
noro+SC-Pdom+AlOH	B1.3	266	0.29			Below Fit Curve Range
noro+SC-Pdom+AlOH	B1.30	260	0.18			Below Fit Curve Range
noro+SC-Pdom/SC-Pdomain+AlOH	B2.1	241	NaN			Below Fit Curve Range
noro+SC-Pdom/SC-Pdomain+AlOH	B2.10	249	NaN			Below Fit Curve Range
noro+SC-Pdom/SC-Pdomain+AlOH	B2.11	251	0.06	0.00	0.0	Below Fit Curve Range
noro+SC-Pdom/SC-Pdomain+AlOH	B2.3	220	NaN			Below Fit Curve Range
noro+SC-Pdom/SC-Pdomain+AlOH	B2.30	264	0.25			Below Fit Curve Range
SpyTag-noro-VLP+AlOH	B3.1	256	0.11			Below Fit Curve Range
SpyTag-noro-VLP+AlOH	B3.10	241	NaN			Below Fit Curve Range
SpyTag-noro-VLP+AlOH	B3.11	255	0.09	0.02	19.7	Below Fit Curve Range
SpyTag-noro-VLP+AlOH	B3.3	265	0.27			Below Fit Curve Range
SpyTag-noro-VLP+AlOH	B3.30	252	0.04			Below Fit Curve Range
SpyCatcher-Pdomain+AlOH	B4.1	255	0.09			Below Fit Curve Range
SpyCatcher-Pdomain+AlOH	B4.10	238	NaN			Below Fit Curve Range
SpyCatcher-Pdomain+AlOH	B4.11	290	0.68	0.05	7.1	Below Fit Curve Range
SpyCatcher-Pdomain+AlOH	B4.3	251	0.02			Below Fit Curve Range
SpyCatcher-Pdomain+AlOH	B4.30	253	0.06			Below Fit Curve Range
TBS+AlOH	B5.1	231	NaN			Below Fit Curve Range
TBS+AlOH	B5.10	226	NaN			Below Fit Curve Range
TBS+AlOH	B5.11	242	NaN	NaN	NaN	Below Fit Curve Range
TBS+AlOH	B5.3	252	0.04			Below Fit Curve Range

IL-4

Group	Mouse code	Raw signal	Calc. Concent	STD DEV concentration	STD DEV concentration/mean concentration (%)	Detection Range
PBS	A1.1	877	NaN			Below Fit Curve Range
PBS	A1.2	1117	0.41			Below Fit Curve Range
PBS	A1.3	929	NaN			Below Fit Curve Range
PBS	A1.30	1018	NaN			Below Fit Curve Range
PBS	A1.4	1008	NaN			Below Fit Curve Range
PBS	A1.5	1065	0.07			Below Fit Curve Range
PBS	A1.6	558	NaN			Below Fit Curve Range
PEED-noro-VLP-SpyCatcher (PCSK9)	A2.10	825	NaN			Below Fit Curve Range
PEED-noro-VLP-SpyCatcher (PCSK9)	A2.11	1005	NaN			Below Fit Curve Range
PEED-noro-VLP-SpyCatcher (PCSK9)	A2.12	1042	NaN			Below Fit Curve Range
PEED-noro-VLP-SpyCatcher (PCSK9)	A2.29	1123	0.45	0.16	36.3	Below Fit Curve Range
PEED-noro-VLP-SpyCatcher (PCSK9)	A2.7	888	NaN			Below Fit Curve Range
PEED-noro-VLP-SpyCatcher (PCSK9)	A2.8	1271	1.36			In Detection Range
PEED-noro-VLP-SpyCatcher (PCSK9)	A2.9	976	NaN			Below Fit Curve Range
SWG-noro-VLP-SpyCatcher(furin)	A3.13	1011	NaN			Below Fit Curve Range
SWG-noro-VLP-SpyCatcher(furin)	A3.14	1056	0.00			Below Fit Curve Range
SWG-noro-VLP-SpyCatcher(furin)	A3.15	1016	NaN			Below Fit Curve Range
SWG-noro-VLP-SpyCatcher(furin)	A3.16	1137	0.54			Below Fit Curve Range
SWG-noro-VLP-SpyCatcher(furin)	A3.17	1050	NaN			Below Fit Curve Range
SWG-noro-VLP-SpyCatcher(furin)	A3.18	1007	NaN	NaN	NaN	Below Fit Curve Range
SpyTag-noro-VLP	A4.19	1024	NaN			Below Fit Curve Range
SpyTag-noro-VLP	A4.20	1045	NaN			Below Fit Curve Range
SpyTag-noro-VLP	A4.21	945	NaN			Below Fit Curve Range
SpyTag-noro-VLP	A4.22	1038	NaN			Below Fit Curve Range
SpyTag-noro-VLP	A4.23	915	NaN			Below Fit Curve Range
SpyTag-noro-VLP	A4.24	1674	3.69			In Detection Range
SpyTag-noro-VLP	A4.28	1088	0.65	0.00	0.0	Below Fit Curve Range
DIIG-noro-VLP-SpyCatcher (PCSK9)	A5.25	1030	NaN			Below Fit Curve Range
DIIG-noro-VLP-SpyCatcher (PCSK9)	A5.31	1066	0.16	0.00	0.0	Below Fit Curve Range
DIIG-noro-VLP-SpyCatcher (PCSK9)	A5.33	1070	0.10			Below Fit Curve Range
DIIG-noro-VLP-SpyCatcher (PCSK9)	A5.34	955	NaN			Below Fit Curve Range
DIIG-noro-VLP-SpyCatcher (PCSK9)	A5.35	1035	NaN			Below Fit Curve Range
DIIG-noro-VLP-SpyCatcher (PCSK9)	A5.36	1063	0.06			Below Fit Curve Range
Pdomain-noro-VLP-SpyCatcher (furin)	A6.27	1043	NaN			Below Fit Curve Range
Pdomain-noro-VLP-SpyCatcher (furin)	A6.37	1038	NaN			Below Fit Curve Range
Pdomain-noro-VLP-SpyCatcher (furin)	A6.38	1139	0.55			Below Fit Curve Range
Pdomain-noro-VLP-SpyCatcher (furin)	A6.39	1013	NaN			Below Fit Curve Range
Pdomain-noro-VLP-SpyCatcher (furin)	A6.40	1125	0.46			Below Fit Curve Range
Pdomain-noro-VLP-SpyCatcher (furin)	A6.41	1052	0.06	0.00	0.0	Below Fit Curve Range
Pdomain-noro-VLP-SpyCatcher (furin)	A6.42	912	NaN			Below Fit Curve Range
noro+SC-Pdom+AlOH	B1.1	733	NaN			Below Fit Curve Range
noro+SC-Pdom+AlOH	B1.10	865	NaN			Below Fit Curve Range
noro+SC-Pdom+AlOH	B1.11	643	NaN	NaN	NaN	Below Fit Curve Range
noro+SC-Pdom+AlOH	B1.3	725	NaN			Below Fit Curve Range
noro+SC-Pdom+AlOH	B1.30	545	NaN			Below Fit Curve Range
noro+SC-Pdom/SC-Pdomain+AlOH	B2.1	777	NaN			Below Fit Curve Range
noro+SC-Pdom/SC-Pdomain+AlOH	B2.10	861	NaN			Below Fit Curve Range
noro+SC-Pdom/SC-Pdomain+AlOH	B2.11	891	NaN	NaN	NaN	Below Fit Curve Range
noro+SC-Pdom/SC-Pdomain+AlOH	B2.3	804	NaN			Below Fit Curve Range
noro+SC-Pdom/SC-Pdomain+AlOH	B2.30	830	NaN			Below Fit Curve Range
SpyTag-noro-VLP+AlOH	B3.1	857	NaN			Below Fit Curve Range
SpyTag-noro-VLP+AlOH	B3.10	851	NaN			Below Fit Curve Range
SpyTag-noro-VLP+AlOH	B3.11	965	NaN	NaN	NaN	Below Fit Curve Range
SpyTag-noro-VLP+AlOH	B3.3	814	NaN			Below Fit Curve Range
SpyTag-noro-VLP+AlOH	B3.30	424	NaN			Below Fit Curve Range
SpyCatcher-Pdomain+AlOH	B4.1	841	NaN			Below Fit Curve Range
SpyCatcher-Pdomain+AlOH	B4.10	941	NaN			Below Fit Curve Range
SpyCatcher-Pdomain+AlOH	B4.11	1145	0.59	0.02	3.7	Below Fit Curve Range
SpyCatcher-Pdomain+AlOH	B4.3	932	NaN			Below Fit Curve Range
SpyCatcher-Pdomain+AlOH	B4.30	851	NaN			Below Fit Curve Range
TBS+AlOH	B5.1	922	NaN			Below Fit Curve Range
TBS+AlOH	B5.10	962	NaN			Below Fit Curve Range
TBS+AlOH	B5.11	983	NaN	NaN	NaN	Below Fit Curve Range
TBS+AlOH	B5.3	980	NaN			Below Fit Curve Range

IL-5

Group	Mouse code	Raw signal	Calc. Concent	STD DEV concentration	STD DEV concentration/mean concentration (%)	Detection Range
PBS	A1.1	556	3.62			In Detection Range
PBS	A1.2	599	4.26			In Detection Range
PBS	A1.3	368	0.88			Below Fit Curve Range
PBS	A1.30	483	2.49			In Detection Range
PBS	A1.4	525	3.15			In Detection Range
PBS	A1.5	603	4.31			In Detection Range
PBS	A1.6	498	2.73			In Detection Range
PEED-noro-VLP-SpyCatcher (PCSK9)	A2.10	557	3.64			In Detection Range
PEED-noro-VLP-SpyCatcher (PCSK9)	A2.11	479	2.42			In Detection Range
PEED-noro-VLP-SpyCatcher (PCSK9)	A2.12	499	2.74			In Detection Range
PEED-noro-VLP-SpyCatcher (PCSK9)	A2.29	603	4.30	0.43	9.97	In Detection Range
PEED-noro-VLP-SpyCatcher (PCSK9)	A2.7	468	2.25			In Detection Range
PEED-noro-VLP-SpyCatcher (PCSK9)	A2.8	569	3.81			In Detection Range
PEED-noro-VLP-SpyCatcher (PCSK9)	A2.9	674	5.33			In Detection Range
SWG-noro-VLP-SpyCatcher(furin)	A3.13	837	7.53			In Detection Range
SWG-noro-VLP-SpyCatcher(furin)	A3.14	513	2.96			In Detection Range
SWG-noro-VLP-SpyCatcher(furin)	A3.15	477	2.39			In Detection Range
SWG-noro-VLP-SpyCatcher(furin)	A3.16	825	7.37			In Detection Range
SWG-noro-VLP-SpyCatcher(furin)	A3.17	632	4.73			In Detection Range
SWG-noro-VLP-SpyCatcher(furin)	A3.18	512	2.95	0.16	5.3	In Detection Range
SpyTag-noro-VLP	A4.19	484	2.51			In Detection Range
SpyTag-noro-VLP	A4.20	459	2.10			In Detection Range
SpyTag-noro-VLP	A4.21	420	1.43			In Detection Range
SpyTag-noro-VLP	A4.22	307	NaN			Below Fit Curve Range
SpyTag-noro-VLP	A4.23	424	1.50			In Detection Range
SpyTag-noro-VLP	A4.24	1656	17.39			In Detection Range
SpyTag-noro-VLP	A4.28	572	3.86	0.22	5.8	In Detection Range
DIIG-noro-VLP-SpyCatcher (PCSK9)	A5.25	594	4.18			In Detection Range
DIIG-noro-VLP-SpyCatcher (PCSK9)	A5.31	596	4.20	0.17	4.0	In Detection Range
DIIG-noro-VLP-SpyCatcher (PCSK9)	A5.33	573	3.87			In Detection Range
DIIG-noro-VLP-SpyCatcher (PCSK9)	A5.34	486	2.54			In Detection Range
DIIG-noro-VLP-SpyCatcher (PCSK9)	A5.35	589	4.11			In Detection Range
DIIG-noro-VLP-SpyCatcher (PCSK9)	A5.36	513	2.96			In Detection Range
Pdomain-noro-VLP-SpyCatcher (furin)	A6.27	547	3.48			In Detection Range
Pdomain-noro-VLP-SpyCatcher (furin)	A6.37	436	1.71			In Detection Range
Pdomain-noro-VLP-SpyCatcher (furin)	A6.38	443	1.83			In Detection Range
Pdomain-noro-VLP-SpyCatcher (furin)	A6.39	501	2.78			In Detection Range
Pdomain-noro-VLP-SpyCatcher (furin)	A6.40	509	2.90			In Detection Range
Pdomain-noro-VLP-SpyCatcher (furin)	A6.41	834	7.49	0.41	5.4	In Detection Range
Pdomain-noro-VLP-SpyCatcher (furin)	A6.42	513	2.96			In Detection Range
noro+SC-Pdom+AlOH	B1.1	535	3.30			In Detection Range
noro+SC-Pdom+AlOH	B1.10	522	3.10			In Detection Range
noro+SC-Pdom+AlOH	B1.11	672	5.30	0.03	0.53	In Detection Range
noro+SC-Pdom+AlOH	B1.3	567	3.78			In Detection Range
noro+SC-Pdom+AlOH	B1.30	578	3.95			In Detection Range
noro+SC-Pdom/SC-Pdomain+AlOH	B2.1	648	4.96			In Detection Range
noro+SC-Pdom/SC-Pdomain+AlOH	B2.10	570	3.83			In Detection Range
noro+SC-Pdom/SC-Pdomain+AlOH	B2.11	591	4.14	0.18	4.4	In Detection Range
noro+SC-Pdom/SC-Pdomain+AlOH	B2.3	420	1.43			In Detection Range
noro+SC-Pdom/SC-Pdomain+AlOH	B2.30	662	5.16			In Detection Range
SpyTag-noro-VLP+AlOH	B3.1	424	1.50			In Detection Range
SpyTag-noro-VLP+AlOH	B3.10	487	2.55			In Detection Range
SpyTag-noro-VLP+AlOH	B3.11	528	3.18	0.05	1.6	In Detection Range
SpyTag-noro-VLP+AlOH	B3.3	899	8.34			In Detection Range
SpyTag-noro-VLP+AlOH	B3.30	612	4.44			In Detection Range
SpyCatcher-Pdomain+AlOH	B4.1	639	4.83			In Detection Range
SpyCatcher-Pdomain+AlOH	B4.10	613	4.46			In Detection Range
SpyCatcher-Pdomain+AlOH	B4.11	785	6.76	0.07	1.0	In Detection Range
SpyCatcher-Pdomain+AlOH	B4.3	744	6.29			In Detection Range
SpyCatcher-Pdomain+AlOH	B4.30	500	2.76			In Detection Range
TBS+AlOH	B5.1	534	3.29			In Detection Range
TBS+AlOH	B5.10	506	2.85			In Detection Range
TBS+AlOH	B5.11	522	3.09	0.16	5.2	In Detection Range
TBS+AlOH	B5.3	462	2.15			In Detection Range

IL-6

Group	Mouse code	Raw signal	Calc. Concent	STD DEV concentration	STD DEV concentration/mean concentration (%)	Detection Range
PBS	A1.1	375	6.82			In Detection Range
PBS	A1.2	408	9.83			In Detection Range
PBS	A1.3	304	NaN			Below Fit Curve Range
PBS	A1.30	331	2.56			Below Detection Range
PBS	A1.4	356	5.03			Below Detection Range
PBS	A1.5	338	3.27			Below Detection Range
PBS	A1.6	366	5.98			In Detection Range
PEED-noro-VLP-SpyCatcher (PCSK9)	A2.10	376	6.91			In Detection Range
PEED-noro-VLP-SpyCatcher (PCSK9)	A2.11	345	3.96			Below Detection Range
PEED-noro-VLP-SpyCatcher (PCSK9)	A2.12	326	2.04			Below Fit Curve Range
PEED-noro-VLP-SpyCatcher (PCSK9)	A2.29	342	3.65	1.19	32.6	Below Detection Range
PEED-noro-VLP-SpyCatcher (PCSK9)	A2.7	373	6.64			In Detection Range
PEED-noro-VLP-SpyCatcher (PCSK9)	A2.8	330	2.46			Below Detection Range
PEED-noro-VLP-SpyCatcher (PCSK9)	A2.9	386	7.84			In Detection Range
SWG-noro-VLP-SpyCatcher(furin)	A3.13	358	5.22			Below Detection Range
SWG-noro-VLP-SpyCatcher(furin)	A3.14	360	5.41			Below Detection Range
SWG-noro-VLP-SpyCatcher(furin)	A3.15	338	3.27			Below Detection Range
SWG-noro-VLP-SpyCatcher(furin)	A3.16	576	24.08			In Detection Range
SWG-noro-VLP-SpyCatcher(furin)	A3.17	351	4.55			Below Detection Range
SWG-noro-VLP-SpyCatcher(furin)	A3.18	368	6.17	0.19	3.0	In Detection Range
SpyTag-noro-VLP	A4.19	326	2.04			Below Fit Curve Range
SpyTag-noro-VLP	A4.20	316	0.96			Below Fit Curve Range
SpyTag-noro-VLP	A4.21	297	NaN			Below Fit Curve Range
SpyTag-noro-VLP	A4.22	287	NaN			Below Fit Curve Range
SpyTag-noro-VLP	A4.23	328	2.25			Below Fit Curve Range
SpyTag-noro-VLP	A4.24	1685	106.42			In Detection Range
SpyTag-noro-VLP	A4.28	326	1.99	0.05	2.6	Below Fit Curve Range
DIIG-noro-VLP-SpyCatcher (PCSK9)	A5.25	526	19.96			In Detection Range
DIIG-noro-VLP-SpyCatcher (PCSK9)	A5.31	342	3.66	0.59	16.2	Below Detection Range
DIIG-noro-VLP-SpyCatcher (PCSK9)	A5.33	314	0.73			Below Fit Curve Range
DIIG-noro-VLP-SpyCatcher (PCSK9)	A5.34	363	5.70			Below Detection Range
DIIG-noro-VLP-SpyCatcher (PCSK9)	A5.35	363	5.70			Below Detection Range
DIIG-noro-VLP-SpyCatcher (PCSK9)	A5.36	345	3.96			Below Detection Range
Pdomain-noro-VLP-SpyCatcher (furin)	A6.27	335	2.97			Below Detection Range
Pdomain-noro-VLP-SpyCatcher (furin)	A6.37	344	3.87			Below Detection Range
Pdomain-noro-VLP-SpyCatcher (furin)	A6.38	337	3.17			Below Detection Range
Pdomain-noro-VLP-SpyCatcher (furin)	A6.39	302	NaN			Below Fit Curve Range
Pdomain-noro-VLP-SpyCatcher (furin)	A6.40	351	4.55			Below Detection Range
Pdomain-noro-VLP-SpyCatcher (furin)	A6.41	364	5.74	0.43	7.4	Below Detection Range
Pdomain-noro-VLP-SpyCatcher (furin)	A6.42	340	3.47			Below Detection Range
noro+SC-Pdom+AlOH	B1.1	329	2.36			Below Detection Range
noro+SC-Pdom+AlOH	B1.10	325	1.94			Below Fit Curve Range
noro+SC-Pdom+AlOH	B1.11	349	4.28	1.51	35.4	Below Detection Range
noro+SC-Pdom+AlOH	B1.3	470	15.24			In Detection Range
noro+SC-Pdom+AlOH	B1.30	386	7.84			In Detection Range
noro+SC-Pdom/SC-Pdomain+AlOH	B2.1	339	3.37			Below Detection Range
noro+SC-Pdom/SC-Pdomain+AlOH	B2.10	369	6.26			In Detection Range
noro+SC-Pdom/SC-Pdomain+AlOH	B2.11	413	10.27	0.00	0.0	In Detection Range
noro+SC-Pdom/SC-Pdomain+AlOH	B2.3	294	NaN			Below Fit Curve Range
noro+SC-Pdom/SC-Pdomain+AlOH	B2.30	347	4.16			Below Detection Range
SpyTag-noro-VLP+AlOH	B3.1	324	1.83			Below Fit Curve Range
SpyTag-noro-VLP+AlOH	B3.10	323	1.72			Below Fit Curve Range
SpyTag-noro-VLP+AlOH	B3.11	359	5.27	0.33	6.3	Below Detection Range
SpyTag-noro-VLP+AlOH	B3.3	307	NaN			Below Fit Curve Range
SpyTag-noro-VLP+AlOH	B3.30	349	4.36			Below Detection Range
SpyCatcher-Pdomain+AlOH	B4.1	325	1.94			Below Fit Curve Range
SpyCatcher-Pdomain+AlOH	B4.10	329	2.36			Below Detection Range
SpyCatcher-Pdomain+AlOH	B4.11	560	22.73	0.53	2.3	In Detection Range
SpyCatcher-Pdomain+AlOH	B4.3	310	0.24			Below Fit Curve Range
SpyCatcher-Pdomain+AlOH	B4.30	328	2.25			Below Fit Curve Range
TBS+AlOH	B5.1	344	3.87			Below Detection Range
TBS+AlOH	B5.10	369	6.26			In Detection Range
TBS+AlOH	B5.11	358	5.17	0.14	2.8	Below Detection Range
TBS+AlOH	B5.3	352	4.65			Below Detection Range

KCGRO

Group	Mouse code	Raw signal	Calc. Concent	STD DEV concentration	STD DEV concentration/mean concentration (%)	Detection Range
PBS	A1.1	9698	39.23			In Detection Range
PBS	A1.2	8033	32.93			In Detection Range
PBS	A1.3	5213	45.65			In Detection Range
PBS	A1.30	10902	43.71			In Detection Range
PBS	A1.4	19380	74.10			In Detection Range
PBS	A1.5	14935	58.38			In Detection Range
PBS	A1.6	10141	40.88			In Detection Range
PEED-noro-VLP-SpyCatcher (PCSK9)	A2.10	15167	59.21			In Detection Range
PEED-noro-VLP-SpyCatcher (PCSK9)	A2.11	9782	39.54			In Detection Range
PEED-noro-VLP-SpyCatcher (PCSK9)	A2.12	14979	58.54			In Detection Range
PEED-noro-VLP-SpyCatcher (PCSK9)	A2.29	13258	52.33	0.04	0.1	In Detection Range
PEED-noro-VLP-SpyCatcher (PCSK9)	A2.7	12170	48.37			In Detection Range
PEED-noro-VLP-SpyCatcher (PCSK9)	A2.8	10691	42.92			In Detection Range
PEED-noro-VLP-SpyCatcher (PCSK9)	A2.9	8519	34.78			In Detection Range
SWG-noro-VLP-SpyCatcher(furin)	A3.13	13339	52.63			In Detection Range
SWG-noro-VLP-SpyCatcher(furin)	A3.14	16486	63.91			In Detection Range
SWG-noro-VLP-SpyCatcher(furin)	A3.15	12944	51.19			In Detection Range
SWG-noro-VLP-SpyCatcher(furin)	A3.16	15878	61.75			In Detection Range
SWG-noro-VLP-SpyCatcher(furin)	A3.17	14424	56.54			In Detection Range
SWG-noro-VLP-SpyCatcher(furin)	A3.18	12689	50.26	0.99	2.0	In Detection Range
SpyTag-noro-VLP	A4.19	9659	39.08			In Detection Range
SpyTag-noro-VLP	A4.20	9578	38.78			In Detection Range
SpyTag-noro-VLP	A4.21	12558	49.79			In Detection Range
SpyTag-noro-VLP	A4.22	173	NaN			Below Fit Curve Range
SpyTag-noro-VLP	A4.23	14139	55.52			In Detection Range
SpyTag-noro-VLP	A4.24	9964	40.22			In Detection Range
SpyTag-noro-VLP	A4.28	11384	45.47	2.95	6.5	In Detection Range
DIIG-noro-VLP-SpyCatcher (PCSK9)	A5.25	24725	92.53			In Detection Range
DIIG-noro-VLP-SpyCatcher (PCSK9)	A5.31	12882	50.96	2.79	5.5	In Detection Range
DIIG-noro-VLP-SpyCatcher (PCSK9)	A5.33	9653	39.06			In Detection Range
DIIG-noro-VLP-SpyCatcher (PCSK9)	A5.34	9548	38.66			In Detection Range
DIIG-noro-VLP-SpyCatcher (PCSK9)	A5.35	9139	37.13			In Detection Range
DIIG-noro-VLP-SpyCatcher (PCSK9)	A5.36	9861	39.83			In Detection Range
Pdomain-noro-VLP-SpyCatcher (furin)	A6.27	12356	49.05			In Detection Range
Pdomain-noro-VLP-SpyCatcher (furin)	A6.37	11130	44.55			In Detection Range
Pdomain-noro-VLP-SpyCatcher (furin)	A6.38	9406	38.13			In Detection Range
Pdomain-noro-VLP-SpyCatcher (furin)	A6.39	10848	43.51			In Detection Range
Pdomain-noro-VLP-SpyCatcher (furin)	A6.40	13106	51.78			In Detection Range
Pdomain-noro-VLP-SpyCatcher (furin)	A6.41	13656	53.77	0.79	1.5	In Detection Range
Pdomain-noro-VLP-SpyCatcher (furin)	A6.42	11610	46.32			In Detection Range
noro+SC-Pdom+AlOH	B1.1	8365	34.20			In Detection Range
noro+SC-Pdom+AlOH	B1.10	9820	39.68			In Detection Range
noro+SC-Pdom+AlOH	B1.11	21337	80.89	0.09	0.1	In Detection Range
noro+SC-Pdom+AlOH	B1.3	25017	93.52			In Detection Range
noro+SC-Pdom+AlOH	B1.30	9051	36.79			In Detection Range
noro+SC-Pdom/SC-Pdomain+AlOH	B2.1	13679	53.86			In Detection Range
noro+SC-Pdom/SC-Pdomain+AlOH	B2.10	12044	47.91			In Detection Range
noro+SC-Pdom/SC-Pdomain+AlOH	B2.11	5997	25.05	1.15	4.6	In Detection Range
noro+SC-Pdom/SC-Pdomain+AlOH	B2.3	12020	47.82			In Detection Range
noro+SC-Pdom/SC-Pdomain+AlOH	B2.30	17795	68.54			In Detection Range
SpyTag-noro-VLP+AlOH	B3.1	16233	63.01			In Detection Range
SpyTag-noro-VLP+AlOH	B3.10	7859	32.27			In Detection Range
SpyTag-noro-VLP+AlOH	B3.11	12299	48.84	0.17	0.3	In Detection Range
SpyTag-noro-VLP+AlOH	B3.3	18228	70.06			In Detection Range
SpyTag-noro-VLP+AlOH	B3.30	9724	39.32			In Detection Range
SpyCatcher-Pdomain+AlOH	B4.1	16097	62.53			In Detection Range
SpyCatcher-Pdomain+AlOH	B4.10	10091	40.69			In Detection Range
SpyCatcher-Pdomain+AlOH	B4.11	13494	53.19	0.19	0.4	In Detection Range
SpyCatcher-Pdomain+AlOH	B4.3	10504	42.23			In Detection Range
SpyCatcher-Pdomain+AlOH	B4.30	17881	68.84			In Detection Range
TBS+AlOH	B5.1	8511	34.75			In Detection Range
TBS+AlOH	B5.10	9132	37.10			In Detection Range
TBS+AlOH	B5.11	11786	46.96	1.49	3.2	In Detection Range
TBS+AlOH	B5.3	15716	61.17			In Detection Range

TNF

Group	Mouse code	Raw signal	Calc. Concent	STD DEV concentration	STD DEV concentration/mean concentration (%)	Detection Range
PBS	A1.1	1717	9.80			In Detection Range
PBS	A1.2	1742	9.97			In Detection Range
PBS	A1.3	869	8.74			In Detection Range
PBS	A1.30	1552	8.73			In Detection Range
PBS	A1.4	1431	7.94			In Detection Range
PBS	A1.5	1305	7.11			In Detection Range
PBS	A1.6	1475	8.23			In Detection Range
PEED-noro-VLP-SpyCatcher (PCSK9)	A2.10	1356	7.44			In Detection Range
PEED-noro-VLP-SpyCatcher (PCSK9)	A2.11	1213	6.50			In Detection Range
PEED-noro-VLP-SpyCatcher (PCSK9)	A2.12	1038	5.34			In Detection Range
PEED-noro-VLP-SpyCatcher (PCSK9)	A2.29	1148	6.07	0.02	0.3	In Detection Range
PEED-noro-VLP-SpyCatcher (PCSK9)	A2.7	1276	6.92			In Detection Range
PEED-noro-VLP-SpyCatcher (PCSK9)	A2.8	1552	8.73			In Detection Range
PEED-noro-VLP-SpyCatcher (PCSK9)	A2.9	1725	9.86			In Detection Range
SWG-noro-VLP-SpyCatcher(furin)	A3.13	1195	6.38			In Detection Range
SWG-noro-VLP-SpyCatcher(furin)	A3.14	1285	6.98			In Detection Range
SWG-noro-VLP-SpyCatcher(furin)	A3.15	1202	6.43			In Detection Range
SWG-noro-VLP-SpyCatcher(furin)	A3.16	1618	9.16			In Detection Range
SWG-noro-VLP-SpyCatcher(furin)	A3.17	1764	10.11			In Detection Range
SWG-noro-VLP-SpyCatcher(furin)	A3.18	1251	6.75	0.63	9.4	In Detection Range
SpyTag-noro-VLP	A4.19	1405	7.77			In Detection Range
SpyTag-noro-VLP	A4.20	1405	7.77			In Detection Range
SpyTag-noro-VLP	A4.21	1229	6.61			In Detection Range
SpyTag-noro-VLP	A4.22	275	NaN			Below Fit Curve Range
SpyTag-noro-VLP	A4.23	1003	5.10			In Detection Range
SpyTag-noro-VLP	A4.24	1320	7.21			In Detection Range
SpyTag-noro-VLP	A4.28	1324	7.23	0.50	6.9	In Detection Range
DIIG-noro-VLP-SpyCatcher (PCSK9)	A5.25	1915	11.09			In Detection Range
DIIG-noro-VLP-SpyCatcher (PCSK9)	A5.31	1603	9.06	1.17	12.9	In Detection Range
DIIG-noro-VLP-SpyCatcher (PCSK9)	A5.33	1357	7.45			In Detection Range
DIIG-noro-VLP-SpyCatcher (PCSK9)	A5.34	1355	7.44			In Detection Range
DIIG-noro-VLP-SpyCatcher (PCSK9)	A5.35	1341	7.35			In Detection Range
DIIG-noro-VLP-SpyCatcher (PCSK9)	A5.36	919	4.54			In Detection Range
Pdomain-noro-VLP-SpyCatcher (furin)	A6.27	1513	8.47			In Detection Range
Pdomain-noro-VLP-SpyCatcher (furin)	A6.37	1300	7.08			In Detection Range
Pdomain-noro-VLP-SpyCatcher (furin)	A6.38	1391	7.68			In Detection Range
Pdomain-noro-VLP-SpyCatcher (furin)	A6.39	1348	7.39			In Detection Range
Pdomain-noro-VLP-SpyCatcher (furin)	A6.40	1200	6.41			In Detection Range
Pdomain-noro-VLP-SpyCatcher (furin)	A6.41	1181	6.29	0.05	0.8	In Detection Range
Pdomain-noro-VLP-SpyCatcher (furin)	A6.42	1005	5.12			In Detection Range
noro+SC-Pdom+AlOH	B1.1	1251	6.75			In Detection Range
noro+SC-Pdom+AlOH	B1.10	1238	6.67			In Detection Range
noro+SC-Pdom+AlOH	B1.11	1568	8.83	0.33	3.7	In Detection Range
noro+SC-Pdom+AlOH	B1.3	1767	10.13			In Detection Range
noro+SC-Pdom+AlOH	B1.30	1722	9.84			In Detection Range
noro+SC-Pdom/SC-Pdomain+AlOH	B2.1	1477	8.24			In Detection Range
noro+SC-Pdom/SC-Pdomain+AlOH	B2.10	1529	8.58			In Detection Range
noro+SC-Pdom/SC-Pdomain+AlOH	B2.11	1342	7.35	0.21	2.9	In Detection Range
noro+SC-Pdom/SC-Pdomain+AlOH	B2.3	1484	8.28			In Detection Range
noro+SC-Pdom/SC-Pdomain+AlOH	B2.30	1399	7.73			In Detection Range
SpyTag-noro-VLP+AlOH	B3.1	1604	9.07			In Detection Range
SpyTag-noro-VLP+AlOH	B3.10	1151	6.09			In Detection Range
SpyTag-noro-VLP+AlOH	B3.11	1529	8.58	0.14	1.6	In Detection Range
SpyTag-noro-VLP+AlOH	B3.3	1746	9.99			In Detection Range
SpyTag-noro-VLP+AlOH	B3.30	1272	6.89			In Detection Range
SpyCatcher-Pdomain+AlOH	B4.1	1459	8.12			In Detection Range
SpyCatcher-Pdomain+AlOH	B4.10	1315	7.17			In Detection Range
SpyCatcher-Pdomain+AlOH	B4.11	1987	11.55	0.40	3.5	In Detection Range
SpyCatcher-Pdomain+AlOH	B4.3	1144	6.04			In Detection Range
SpyCatcher-Pdomain+AlOH	B4.30	1630	9.24			In Detection Range
TBS+AlOH	B5.1	1244	6.71			In Detection Range
TBS+AlOH	B5.10	1675	9.53			In Detection Range
TBS+AlOH	B5.11	1647	9.34	0.57	6.1	In Detection Range
TBS+AlOH	B5.3	1391	7.68			In Detection Range
